# A Novel Version of the Arcsine–Rayleigh Distribution with Entropy Measures, Statistical Inference, and Applications

**DOI:** 10.3390/e28040464

**Published:** 2026-04-17

**Authors:** Asmaa S. Al-Moisheer, Khalaf S. Sultan, Moustafa N. Mousa, Mahmoud M. M. Mansour

**Affiliations:** 1 Department of Mathematics and Statistics, Faculty of Science, Imam Mohammad Ibn Saud Islamic University (IMSIU), Riyadh 11432, Saudi Arabia; asAlMoisheer@imamu.edu.sa; 2 Mathematics Department, Faculty of Science, Al-Azhar University, Nasr City, Cairo 11884, Egypt; ksultan@azhar.edu.eg; 3 Department of Mathematics, Faculty of Science, Kafrelsheikh University, Kafrelsheikh 33516, Egypt; mostafa.nabil_a006@sci.kfs.edu.eg; 4 Department of Basic Science, Faculty of Engineering, The British University in Egypt, El Sherouk City, Cairo 11837, Egypt

**Keywords:** Unit Arcsine–Rayleigh distribution, bounded data modeling, reliability analysis, entropy measures, maximum likelihood, maximum product spacing, Bayesian inference

## Abstract

This paper presents a new distribution on the unit interval, named the Unit Arcsine–Rayleigh distribution (UASRD), which is the result of the exponential transformation of the Arcsine–Rayleigh distribution. The model suggested is versatile and can be used in modeling limited reliability and proportion data. Entropy-based measures are also studied to determine the uncertainty and information content of the proposed model and further explain the probabilistic nature of the proposed model and its potential applicability in information-theoretic and reliability tasks. These findings demonstrate the utility of the suggested model in the study of the limited data in the context of information theory. Basic statistical characteristics are derived, such as cumulative and density functions, quantile function, reliability and hazard functions, and ordinary moments. Estimation of parameters is obtained through approaches of maximum likelihood and maximum product spacing and Bayesian estimation of parameters. The performance of the estimators is also assessed by a Monte Carlo simulation study, and the application of real data shows the utility of the proposed model to the analysis of bounded data.

## 1. Motivation and Introduction

Modeling random variables supported on the unit interval (0,1) remains a central topic in modern statistical inference. Such data arise naturally as proportions, rates, probabilities, indices, and normalized measurements in reliability engineering, economics, environmental studies, medicine, and the social sciences. Because the support is intrinsically bounded, conventional distributions on (0,∞) or (−∞,∞) are generally inadequate without transformation. This has motivated sustained interest in flexible unit distributions with interpretable parameters and convenient inferential procedures.

The beta distribution [1] is the classical benchmark for modeling unit data due to its shape flexibility. However, likelihood-based analysis for beta-type models may involve special functions, which can complicate numerical optimization and the derivation of certain analytical properties. Consequently, several alternatives have been proposed, including the Kumaraswamy [2], Topp–Leone [3], and power-type families [4], as well as many extensions. In recent years, transformation-based construction has become particularly influential, since it provides a systematic way to transfer structural properties from nonnegative lifetime models to unit-support counterparts.

Among these constructions, the exponential mapping $Z=e^{-X}$, where *X* is nonnegative, has proven especially useful. This transformation yields closed-form unit distributions while preserving substantial shape flexibility for the density and hazard functions. Some well-known examples are the unit inverse Gaussian [5], unit Lindley [6], unit Weibull [7], unit Gompertz [8], unit gamma [9], unit inverse Maxwell–Boltzmann [10], unit-modified Weibull [11], unit exponential delay time [12], and unit J-shaped [13]. A related model is the Unit Arcsine-Exponential Distribution (UASED), introduced in [14]. Although both the UASED and the proposed model are obtained through exponential transformation, the present construction is generated from the *Arcsine-Rayleigh distribution* (ASRD), whereas the UASED is based on the *Arcsine-Exponential distribution* (ASED); see [15]. This difference in the parent lifetime model leads to a distinct unit distribution with different structural and inferential characteristics.

Within this framework, the ASRD is an appealing baseline lifetime model because of its parsimonious one-parameter form and analytically tractable characteristics. Let X∼ASRD(ζ), ζ>0. Its cumulative distribution function (CDF) and probability density function (PDF) are given by

(1) $$F_{X}\left(x;\zeta \right)=\frac{2}{\pi }\;\arcsin\;\left(\sqrt{1-e^{-\frac{x^{2}}{2\zeta^{2}}}}\right),\;x>0,\;\zeta >0,$$

and

(2) $$f_{X}\left(x;\zeta \right)=\frac{x\sqrt{e^{-\frac{x^{2}}{2\zeta^{2}}}}}{\pi \zeta^{2}\sqrt{1-e^{-\frac{x^{2}}{2\zeta^{2}}}}},\;x>0,\;\zeta >0.$$

Although the ASRD is effective for modeling nonnegative lifetime data, its unbounded support makes it unsuitable for direct use with proportion-type observations. To address this issue, we define the *Unit Arcsine–Rayleigh distribution* (UASRD) via the transformation $Z=e^{-X}$, where X∼ASRD(ζ). The resulting model is supported on (0,1), retains analytical simplicity, and accommodates diverse density and hazard-rate shapes with a single parameter.

Entropy measures are also useful for describing the uncertainty and information content associated with bounded random variables. In particular, Shannon entropy and its generalizations provide quantitative tools for assessing randomness and informational variability within a distribution. For the proposed UASRD, the study of entropy measures complements the classical analysis based on moments and reliability characteristics, thereby providing a broader understanding of the probabilistic structure of the model.

The present work, therefore, contributes to the growing class of unit distributions by introducing a new bounded model with tractable mathematical properties, feasible inferential procedures, and potential practical relevance.

The main contributions of this paper are summarized as follows:

(i) We propose a new one-parameter unit distribution generated from the ASRD via exponential transformation.
(ii) We derive key distributional properties, including closed-form expressions for the CDF/PDF, quantile function, moments, and several entropy measures.
(iii) We develop frequentist and Bayesian inference procedures, and we evaluate their finite-sample behavior through Monte Carlo experiments.
(iv) We demonstrate the practical utility of the proposed model through applications to real datasets arising from reliability and proportion-data settings.

The remainder of the article is organized as follows. Section 2 defines the UASRD and presents its fundamental functions. Section 3 studies distributional characteristics and entropy-related quantities. Section 4 develops frequentist estimation based on likelihood and spacing methods, together with asymptotic interval estimation. Section 5 presents Bayesian inference under informative and non-informative priors using the Metropolis–Hastings algorithm. Section 6 reports simulation results, and Section 7 provides real-data illustrations. Concluding remarks are given in Section 8.

## 2. Unit Arcsine–Rayleigh Distribution

Let X∼ASRD(ζ) with CDF as in Equation (1). To construct a unit-bounded distribution from the ASRD, we apply the monotone decreasing transformation $Z=e^{-X}$. Since X∈(0,∞), it follows that Z∈(0,1), and the inverse transformation is given by X=−logZ. Then the CDF of *Z* can be derived as

(3) $$\begin{array}{cc} F_{Z}\left(z;\zeta \right) & =P\left(Z\leq z\right)=P\left(e^{-X}\leq z\right)=P\left(X\geq -\log z\right)=1-F_{X}\left(-\log z;\zeta \right)\\ \; & =1-\frac{2}{\pi }\;\arcsin\;\left(\sqrt{1-e^{-\frac{{\left(-\log z\right)}^{2}}{2\zeta^{2}}}}\right)\\ \; & =1-\frac{2}{\pi }\;\arcsin\;\left(\sqrt{1-e^{-\frac{\log^{2}\left(z\right)}{2\zeta^{2}}}}\right),\;0<z<1,\;\zeta >0. \end{array}$$

Consequently, the transformed random variable *Z* is said to follow a UASRD, denoted by Z∼UASRD(ζ), with CDF given in Equation (3). The corresponding PDF is given by

(4) $$f_{Z}\left(z;\zeta \right)=-\frac{\log z\;e^{-\frac{\log^{2}\left(z\right)}{4\zeta^{2}}}}{\pi \zeta^{2}z\sqrt{1-e^{-\frac{\log^{2}\left(z\right)}{2\zeta^{2}}}}},\;0<z<1,\;\zeta >0.$$

**Figure 1:**
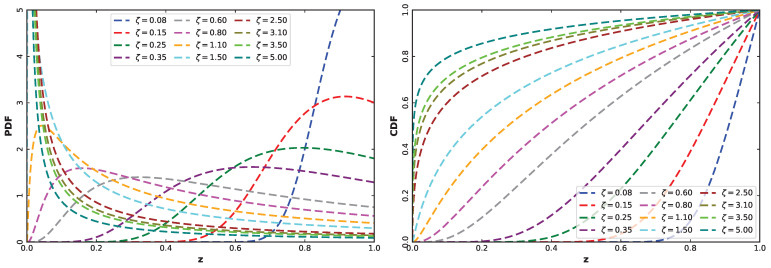
Plots of the PDF (**left**) and CDF (**right**) at given values of the shape parameter ζ.

Differentiating the CDF in Equation (3) with respect to *z* yields the PDF in Equation (4), which demonstrates the fact that the UASRD has a closed-form density. The PDF and CDF of the model can be seen to be flexible on the unit interval, which is shown in Figure 1. The PDF can be monotonic (decreasing or increasing) or unimodal with different skewness, and the CDF can be either concave, convex, or sigmoidal depending on the value of *z*. The flexibility of the given model in the domain of (0,1) is emphasized by this variety of shapes.

The reliability function (RF) of the random variable *Z* and associated hazard function (HF) are, respectively, as follows:

(5) $$R_{Z}\left(z;\zeta \right)=1-F_{Z}\left(z;\zeta \right)=\frac{2}{\pi }\;\arcsin\;\left(\sqrt{1-e^{-\frac{\log^{2}\left(z\right)}{2\zeta^{2}}}}\right),\;0<z<1,$$

and

(6) $$h_{Z}\left(z;\zeta \right)=\frac{f_{Z}\left(z;\zeta \right)}{R_{Z}\left(z;\zeta \right)}=-\frac{\log\left(z\right)\sqrt{e^{-\frac{\log^{2}\left(z\right)}{2\zeta^{2}}}}}{2\zeta^{2}z\sqrt{1-e^{-\frac{\log^{2}\left(z\right)}{2\zeta^{2}}}}\arcsin\left(\sqrt{1-e^{-\frac{\log^{2}\left(z\right)}{2\zeta^{2}}}}\right)},\;0<z<1.$$

**Figure 2:**
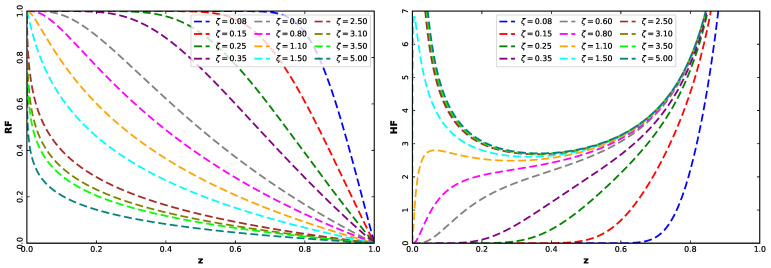
RF (**left**) and HF (**right**) of the UASRD for various values of ζ.

The UASRD has a large variety of representative forms of the RF and HF, as illustrated in Figure 2. The RF monotonically decreases and can be concave, convex, or almost linear with the value of ζ. Conversely, the HF is highly flexible, assuming the shapes of increasing, decreasing, bathtub-shaped, or U-shaped. This flexibility is especially relevant in reliability analysis, as it allows the model to represent various phases of failure rate with such aspects as infant mortality, a phase with a constant risk, and wear-out behavior within the unit interval.

## 3. Statistical Properties of the UASRD

This section focuses on the derivation of important mathematical properties of the proposed UASRD.

### 3.1. Identifiability Property and Mode

This subsection focuses on the identifiability property and the mode of the UASRD. The shape parameter ζ is considered *identifiable* if equality of the CDFs necessarily implies equality of the corresponding parameters. The mode, representing the value at which the PDF attains its maximum, characterizes the boundary behavior of the distribution and depends solely on ζ.

**Theorem** **1** (Identifiability of the UASRD Parameter)**.** *Let Z∼UASRD(ζ) with CDF $F_{Z}\left(z;\zeta \right)$ given in Equation (3). Then the parameter ζ>0 is identifiable, that is, $F_{Z}\left(z;\zeta_{1}\right)=F_{Z}\left(z;\zeta_{2}\right)$ for all z∈(0,1) implies that $\zeta_{1}=\zeta_{2}.$*

**Proof.**  Suppose that two parameters $\zeta_{1}$ and $\zeta_{2}$ produce the same CDF:

$$\begin{array}{cc} F_{Z}\left(z;\zeta_{1}\right) & =F_{Z}\left(z;\zeta_{2}\right),\\ 1-\frac{2}{\pi }\;\arcsin\;\left(\sqrt{1-e^{-\frac{\log^{2}\left(z\right)}{2\zeta_{1}^{2}}}}\right)\; & =1-\frac{2}{\pi }\;\arcsin\;\left(\sqrt{1-e^{-\frac{\log^{2}\left(z\right)}{2\zeta_{2}^{2}}}}\right),\\ \arcsin\;\left(\sqrt{1-e^{-\frac{\log^{2}\left(z\right)}{2\zeta_{1}^{2}}}}\right)\; & =\arcsin\;\left(\sqrt{1-e^{-\frac{\log^{2}\left(z\right)}{2\zeta_{2}^{2}}}}\right),\\ \sqrt{1-e^{-\frac{\log^{2}\left(z\right)}{2\zeta_{1}^{2}}}}\; & =\sqrt{1-e^{-\frac{\log^{2}\left(z\right)}{2\zeta_{2}^{2}}}},\\ 1-e^{-\frac{\log^{2}\left(z\right)}{2\zeta_{1}^{2}}}\; & =1-e^{-\frac{\log^{2}\left(z\right)}{2\zeta_{2}^{2}}},\\ e^{-\frac{\log^{2}\left(z\right)}{2\zeta_{1}^{2}}}\; & =e^{-\frac{\log^{2}\left(z\right)}{2\zeta_{2}^{2}}},\\ -\frac{\log^{2}\left(z\right)}{2\zeta_{1}^{2}}\; & =-\frac{\log^{2}\left(z\right)}{2\zeta_{2}^{2}},\;0<z<1. \end{array}$$

Because $\log^{2}\left(z\right)>0$ for all z∈(0,1), we obtain $\zeta_{1}^{2}=\zeta_{2}^{2}$. Since $\zeta_{1},\zeta_{2}>0$, it follows that $\zeta_{1}=\zeta_{2}$. Thus, the UASRD is identifiable with respect to the parameter ζ.    □

**Theorem** **2** (Mode of the UASRD)**.** *Let Z∼UASRD(ζ) with PDF $f_{Z}\left(z;\zeta \right)$ given in Equation (4). Then the mode $z_{\mathrm{mode}}\left(\zeta \right)\in \left(0,1\right)$ is characterized as a solution of the non-linear equation*

(7) $$\left(1-\log z\right)\left(1-e^{-\frac{\log^{2}\left(z\right)}{2\zeta^{2}}}\right)-\frac{\log^{2}\left(z\right)}{2\zeta^{2}}=0,\;0<z<1,\;\zeta >0.$$

**Proof.**  Since $f_{Z}\left(z;\zeta \right)>0$ for all z∈(0,1), maximizing $f_{Z}$ is equivalent to maximizing its log-density. From Equation (4), the log-density is given by

(8) $$\begin{array}{cc} \log f_{Z}\left(z;\zeta \right) & =\log\left(-\log z\right)-\log\left(\pi \zeta^{2}\right)-\log z-\frac{\log^{2}\left(z\right)}{4\zeta^{2}}-\frac{1}{2}\log\;\left(1-e^{-\frac{\log^{2}\left(z\right)}{2\zeta^{2}}}\right). \end{array}$$

Differentiating Equation (8) with respect to *z* and setting the derivative equal to zero yields the critical point equation. Multiplying both sides by zlogz and simplifying, we obtain

$$1-\log z-\frac{\log^{2}\left(z\right)}{2\zeta^{2}}\left(1+\frac{e^{-\frac{\log^{2}\left(z\right)}{2\zeta^{2}}}}{1-e^{-\frac{\log^{2}\left(z\right)}{2\zeta^{2}}}}\right)=0.$$

Further simplification leads to Equation (7). This equation is non-linear and does not admit a closed-form solution in terms of elementary functions. Hence, the mode $z_{\mathrm{mode}}\left(\zeta \right)$ is obtained numerically, for a given ζ, using root-finding methods such as the Newton–Raphson algorithm.    □

### 3.2. Quantile Function and Random Sample Generation

This subsection focuses on deriving the quantile function (QF) and using it to simulate random samples from the proposed UASRD.

**Theorem** **3** (Quantile Function of the UASRD)**.** *Let Z∼UASRD(ζ) with CDF $F_{Z}\left(z;\zeta \right)$ given in Equation (3). Then, for 0<q<1, the QF of Z, defined by $Q_{Z}\left(q;\zeta \right)=F_{Z}^{-1}\left(q;\zeta \right)$, is given by*

(9) $$Q_{Z}\left(q;\zeta \right)=e^{-\zeta \sqrt{-2\log\;\left(\sin^{2}\;\left(\frac{\pi }{2}q\right)\right)}}=e^{-2\zeta \sqrt{\log\left(\csc\left(\frac{\pi }{2}q\right)\right)}}.$$

**Proof.**  By definition, the QF is the inverse of the CDF, that is,

$$Q_{Z}\left(q;\zeta \right)=F_{Z}^{-1}\left(q;\zeta \right),\;0<q<1.$$

Let $F_{Z}\left(z;\zeta \right)=q$. From Equation (3), we have

$$1-\frac{2}{\pi }\;\arcsin\;\left(\sqrt{1-e^{-\frac{\log^{2}\left(z\right)}{2\zeta^{2}}}}\right)=q,\;\mathrm{so}\;\mathrm{that}\;\arcsin\;\left(\sqrt{1-e^{-\frac{\log^{2}\left(z\right)}{2\zeta^{2}}}}\right)=\frac{\pi }{2}-\frac{\pi }{2}q.$$

Applying the sine function, then squaring both sides and rearranging, we obtain

$$\sqrt{1-e^{-\frac{\log^{2}\left(z\right)}{2\zeta^{2}}}}=\cos\;\left(\frac{\pi }{2}q\right),\;\mathrm{so}\;\mathrm{that}\;e^{-\frac{\log^{2}\left(z\right)}{2\zeta^{2}}}=1-\cos^{2}\;\left(\frac{\pi }{2}q\right)=\sin^{2}\;\left(\frac{\pi }{2}q\right).$$

Taking the natural logarithm of both sides, we obtain

$$-\frac{\log^{2}\left(z\right)}{2\zeta^{2}}=\log\left(\sin^{2}\;\left(\frac{\pi }{2}q\right)\right),\;\mathrm{so}\;\mathrm{that}\;\log^{2}\left(z\right)=-2\zeta^{2}\log\left(\sin^{2}\;\left(\frac{\pi }{2}q\right)\right).$$

Taking square roots yields

$$\log z=\pm \zeta \sqrt{-2\log\left(\sin^{2}\;\left(\frac{\pi }{2}q\right)\right)}.$$

Since 0<z<1, it follows that logz<0, and therefore the negative branch must be selected. Hence,

$$\log z=-\zeta \sqrt{-2\log\left(\sin^{2}\;\left(\frac{\pi }{2}q\right)\right)}.$$

Exponentiating both sides gives

$$z=e^{-\zeta \sqrt{-2\log\;\left(\sin^{2}\;\left(\frac{\pi }{2}q\right)\right)}},\;\mathrm{or}\;\mathrm{equivalently}\;z=e^{-2\zeta \sqrt{\log\left(\csc\left(\frac{\pi }{2}q\right)\right)}},$$

which establishes the result.    □

**Remark** **1** (Quartiles and Median of the UASRD)**.** *For the UASRD, the first quartile ($Q_{1}$), median ($Q_{2}$), and third quartile ($Q_{3}$) correspond to the 25th, 50th, and 75th percentiles of the distribution, respectively. These can be obtained directly from the QF by setting q=0.25, 0.50, and 0.75:*

$$Q_{1}=Q_{Z}\left(0.25;\zeta \right),\;Q_{2}=Q_{Z}\left(0.50;\zeta \right),\;\mathrm{and}\;Q_{3}=Q_{Z}\left(0.75;\zeta \right).$$

**Remark** **2** (Random Sample Generation from the UASRD)**.** *A random variable Z∼UASRD(ζ) is generated by applying the inverse of the CDF defined in Equation (9), where U∼Uniform(0,1). Accordingly, a random sample $\left\{Z_{1},\ldots ,Z_{n}\right\}$ is obtained from the UASRD(ζ) through the following steps:*

***Step 1:***  *Generate $U_{1},\ldots ,U_{n}\overset{i.i.d.}{∼}\mathrm{Uniform}\left(0,1\right)$;*
***Step 2:***  *Compute $Z_{i}=e^{-\zeta \sqrt{-2\log\;\left(\sin^{2}\;\left(\frac{\pi }{2}U_{i}\right)\right)}},\;i=1,\ldots ,n.$*

*This approach is computationally efficient for use in Monte Carlo simulations, bootstrap procedures, and parameter estimation methods.*

### 3.3. Moments and Related Statistical Quantities

This subsection focuses on obtaining the moments, central moments, moment generating function, characteristic function, cumulant generating function, and incomplete moments for the proposed UASRD, as well as the computation and discussion of key associated measures: mean, variance, skewness, kurtosis, and coefficient of variation.

**Theorem** **4** (Moments of the UASRD)**.** *Let Z∼UASRD(ζ) with PDF $f_{Z}\left(z;\zeta \right)$ given in Equation (4), where ζ>0. Then, for any real m>−1, the $m^{\mathrm{th}}$ non-central moment of Z exists and is given by*

(10) μm′=E[Zm]=∑k=0∞2kk4k2π(2k+1)−2mζπ(2k+1)3/2em2ζ22k+1erfcmζ2k+1.

**Proof.**  By definition and using Equation (4), we have

$$\mu_{m}^{\prime }=E\left[Z^{m}\right]=\int_{0}^{1}z^{m}f_{Z}\left(z;\zeta \right)\;dz=\frac{1}{\pi \zeta^{2}}\int_{0}^{1}\frac{-z^{m-1}\log\left(z\right)\;e^{-\frac{\log^{2}\left(z\right)}{4\zeta^{2}}}}{\sqrt{1-e^{-\frac{\log^{2}\left(z\right)}{2\zeta^{2}}}}}\;dz.$$

Set y=−logz so that $z=e^{-y}$ and $dz=-e^{-y}dy$. As *z* increases from 0 to 1, *y* decreases from ∞ to 0, and hence

$$\mu_{m}^{\prime }=\frac{1}{\pi \zeta^{2}}\int_{0}^{\infty }\frac{y\;e^{-my}\;e^{-\frac{y^{2}}{4\zeta^{2}}}}{\sqrt{1-e^{-\frac{y^{2}}{2\zeta^{2}}}}}\;dy.$$

For y>0, define $w=e^{-\frac{y^{2}}{2\zeta^{2}}}\in \left(0,1\right)$. Using the binomial expansion

11−w=∑k=0∞2kk4kwk,|w|<1,weobtain11−e−y22ζ2=∑k=0∞2kk4ke−ky22ζ2.

Since the integrand is nonnegative for m>−1, Tonelli’s theorem justifies interchanging summation and integration, yielding

μm′=1πζ2∑k=0∞2kk4k∫0∞ye−my−(2k+1)y24ζ2dy.

Recognizing that this integral is a standard Gaussian-type integral, given by

$$\int_{0}^{\infty }y\;e^{-ay^{2}-my}\;dy=\frac{1}{2a}-\frac{m\sqrt{\pi }}{4a^{3/2}}e^{\frac{m^{2}}{4a}}\;\mathrm{erfc}\;\left(\frac{m}{2\sqrt{a}}\right),\;a>0,\;m>0,$$

where erfc(·) is the complementary error function. With $a=\frac{2k+1}{4\zeta^{2}}$, we obtain

$$\int_{0}^{\infty }y\;e^{-my-\frac{\left(2k+1\right)y^{2}}{4\zeta^{2}}}\;dy=\frac{2\zeta^{2}}{2k+1}-\frac{2m\sqrt{\pi }\;\zeta^{3}}{{\left(2k+1\right)}^{3/2}}e^{\frac{m^{2}\zeta^{2}}{2k+1}}\mathrm{erfc}\;\left(\frac{m\zeta }{\sqrt{2k+1}}\right).$$

Substituting into the series representation of $\mu_{m}^{\prime }$ and simplifying gives Equation (10). This completes the proof.    □

**Theorem** **5** (Central Moments of the UASRD)**.** *Let Z∼UASRD(ζ) with PDF $f_{Z}\left(z;\zeta \right)$ given in Equation (4), and let $\mu =E\left[Z\right]=\mu_{1}^{\prime }$ denote the mean. Then, for every integer m≥1, the $m^{\mathrm{th}}$ central moment $\mu_{m}=E\;\left[{\left(Z-\mu \right)}^{m}\right]$ exists and is given by*

(11) μm=∑j=0mmj(−μ)m−j∑k=0∞2kk4k2π(2k+1)−2jζπ(2k+1)3/2ej2ζ22k+1erfcjζ2k+1.

**Proof.**  By definition, the $m^{th}$ central moment of *Z* is

$$\mu_{m}=E\left[{\left(Z-\mu \right)}^{m}\right]=\int_{0}^{1}{\left(z-\mu \right)}^{m}f_{Z}\left(z;\zeta \right)\;dz.$$

Expanding ${\left(z-\mu \right)}^{m}$ using the binomial theorem gives (z−μ)m=∑j=0mmj(−μ)m−jzj. Substituting this expansion into the integral yields

μm=∫01∑j=0mmj(−μ)m−jzjfZ(z;ζ)dz=∑j=0mmj(−μ)m−j∫01zjfZ(z;ζ)dz=∑j=0mmj(−μ)m−jμj′.

Substituting the expression for the $j^{th}$ non-central moment from Equation (10) into the series representation of $\mu_{m}$ yields Equation (11), which completes the proof.    □

**Corollary** **1** (Descriptive Measures of the UASRD)**.** *Let Z∼UASRD(ζ) with ζ>0. Then, its main descriptive measures are given as follows:*

***1.***  *The mean, given by $\mu =E\left[Z\right]=\mu_{1}^{\prime }$, is expressed as*
  μ=∑k=0∞2kk4k2π(2k+1)−2ζπ(2k+1)3/2eζ22k+1erfcζ2k+1.
***2.***  *The variance, given by $\sigma^{2}=\mathrm{Var}\left(Z\right)=\mu_{2}=\mu_{2}^{\prime }-\mu^{2}$, is obtained by*
  σ2=∑k=0∞2kk4k2π(2k+1)−4ζπ(2k+1)3/2e4ζ22k+1erfc2ζ2k+1−∑k=0∞2kk4k2π(2k+1)−2ζπ(2k+1)3/2eζ22k+1erfcζ2k+12.
***3.***  *The skewness, given by $\left(\gamma_{1}=\frac{\mu_{3}}{\mu_{2}^{3/2}}=\frac{\mu_{3}^{\prime }-3\mu \;\mu_{2}^{\prime }+2\mu^{3}}{{\left(\mu_{2}^{\prime }-\mu^{2}\right)}^{3/2}}\right)$, is expressed as*
  γ1={∑k=0∞2kk4k2π(2k+1)−6ζπ(2k+1)3/2e9ζ22k+1erfc3ζ2k+1−3μ∑k=0∞2kk4k2π(2k+1)−4ζπ(2k+1)3/2e4ζ22k+1erfc2ζ2k+1+2μ3}×∑k=0∞2kk4k2π(2k+1)−4ζπ(2k+1)3/2e4ζ22k+1erfc2ζ2k+1−μ2−32.
***4.***  *The kurtosis, given by $\left(\gamma_{2}=\frac{\mu_{4}}{\mu_{2}^{2}}-3=\frac{\mu_{4}^{\prime }-4\mu \;\mu_{3}^{\prime }+6\mu^{2}\mu_{2}^{\prime }-3\mu^{4}}{{\left(\mu_{2}^{\prime }-\mu^{2}\right)}^{2}}-3\right)$, is obtained by*
  γ2={∑k=0∞2kk4k2π(2k+1)−8ζπ(2k+1)3/2e16ζ22k+1erfc4ζ2k+1−4μ∑k=0∞2kk4k2π(2k+1)−6ζπ(2k+1)3/2e9ζ22k+1erfc3ζ2k+1+6μ2∑k=0∞2kk4k2π(2k+1)−4ζπ(2k+1)3/2e4ζ22k+1erfc2ζ2k+1−3μ4}×∑k=0∞2kk4k2π(2k+1)−4ζπ(2k+1)3/2e4ζ22k+1erfc2ζ2k+1−μ2−2−3.
***5.***  *The coefficient of variation, given by $\left(\mathrm{CV}=\frac{\sigma }{\mu }\right)$, is expressed as*
  CV=μ−2∑k=0∞2kk4k2π(2k+1)−4ζπ(2k+1)3/2e4ζ22k+1erfc2ζ2k+1−1.
***6.***  *The coefficient of dispersion, given by $\left(\mathrm{CD}=\frac{\sigma^{2}}{\mu }=\frac{\mu_{2}^{\prime }-\mu^{2}}{\mu }\right)$, is obtained by*
  CD=μ−1∑k=0∞2kk4k2π(2k+1)−4ζπ(2k+1)3/2e4ζ22k+1erfc2ζ2k+1−μ.

**Figure 3:**
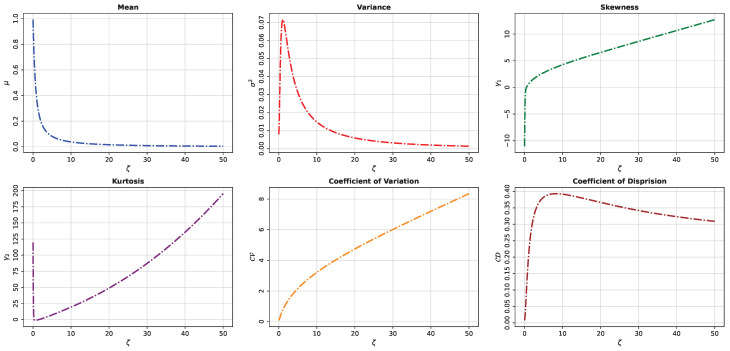
Statistical properties of the UASRD as a function of the shape parameter ζ.

Some numerical values of the quartiles $Q_{1}$, $Q_{2}$, and $Q_{3}$, together with μ, $\sigma^{2}$, $\gamma_{1}$, $\gamma_{2}$, CV, and CD, for various values of the shape parameter ζ of the UASRD are reported in Table 1. The corresponding behavior of these measures as functions of ζ is displayed in Figure 3, illustrating the evolution of the distributional characteristics as ζ increases. From the results, the following observations can be made:

1. $Q_{1}$, $Q_{2}$, $Q_{3}$, and μ decrease monotonically as ζ increases, indicating that the distribution progressively concentrates its mass toward the origin.
2. $\sigma^{2}$ exhibits a unimodal pattern; it increases initially, attains a maximum around ζ≈1.12, and then decreases for larger values of ζ.
3. CV increases steadily with ζ, while CD increases rapidly for small values of ζ, reaches a maximum near ζ≈8.60, and subsequently decreases gradually.
4. $\gamma_{1}$ increases from negative to positive values, indicating a transition from left-skewness to right-skewness as ζ increases.
5. $\gamma_{2}$ initially decreases for small values of ζ and then increases sharply, reflecting a change in tail behavior and peakedness, with heavier tails emerging for larger ζ.
6. Overall, the UASRD demonstrates considerable flexibility, transitioning from a highly concentrated and left-skewed distribution at small values of ζ to a more dispersed, right-skewed, and heavy-tailed distribution as ζ increases.

**Theorem** **6** (Moment Generating Function of the UASRD)**.** *Let Z∼UASRD(ζ) with PDF $f_{Z}\left(z;\zeta \right)$ given in Equation (4), where ζ>0. Then, for every t∈R, the moment generating function (MGF) of Z exists and is given by*

(12) MZ(t)=EetZ=∑m=0∞tmm!∑k=0∞2kk4k2π(2k+1)−2mζπ(2k+1)3/2em2ζ22k+1erfcmζ2k+1.

**Proof.**  By definition and using Equation (4), the MGF of *Z* is

$$M_{Z}\left(t\right)=E\left[e^{tZ}\right]=\int_{0}^{1}e^{tz}\;f_{Z}\left(z;\zeta \right)\;dz.$$

Using the power-series expansion $e^{tz}=\sum_{m=0}^{\infty }\frac{t^{m}}{m!}z^{m}$, which converges absolutely for all t∈R and 0<z<1, we obtain

$$M_{Z}\left(t\right)=\int_{0}^{1}\left(\sum_{m=0}^{\infty }\frac{t^{m}}{m!}z^{m}\right)f_{Z}\left(z;\zeta \right)\;dz.$$

By interchanging summation and integration, we obtain

$$M_{Z}\left(t\right)=\sum_{m=0}^{\infty }\frac{t^{m}}{m!}\int_{0}^{1}z^{m}f_{Z}\left(z;\zeta \right)\;dz=\sum_{m=0}^{\infty }\frac{t^{m}}{m!}\;\mu_{m}^{\prime },$$

where $\mu_{m}^{\prime }=E\left[Z^{m}\right]$ denotes the $m^{\mathrm{th}}$ non-central moment of *Z*. Substituting the explicit series representation of $\mu_{m}^{\prime }$ from Equation (10) yields (12), completing the proof.    □

**Remark** **3.** *The $m^{th}$ non-central moment of Z can be obtained from the MGF $M_{Z}\left(t\right)$ as follows*

$$\mu_{m}^{\prime }={\left.\frac{d^{m}}{dt^{m}}M_{Z}\left(t\right)\right|}_{t=0},\;m=1,2,\ldots .$$

**Theorem** **7** (Characteristic Function of the UASRD)**.** *Let Z∼UASRD(ζ) with PDF $f_{Z}\left(z;\zeta \right)$ given in Equation (4), where ζ>0. Then, for every t∈R, the characteristic function (CF) of Z exists and is given by*

(13) φZ(t)=EeitZ=∑m=0∞(it)mm!∑k=0∞2kk4k2π(2k+1)−2mζπ(2k+1)3/2em2ζ22k+1erfcmζ2k+1.

**Proof.**  By definition and using Equation (4), the CF of *Z* is

$$\varphi_{Z}\left(t\right)=E\left[e^{itZ}\right]=\int_{0}^{1}e^{itz}\;f_{Z}\left(z;\zeta \right)\;dz,\;i=\sqrt{-1}.$$

Using the absolutely convergent power-series expansion $e^{itz}=\sum_{m=0}^{\infty }\frac{{\left(it\right)}^{m}}{m!}z^{m}$ for all t∈R and 0<z<1, we have

$$\varphi_{Z}\left(t\right)=\int_{0}^{1}\left(\sum_{m=0}^{\infty }\frac{{\left(it\right)}^{m}}{m!}z^{m}\right)f_{Z}\left(z;\zeta \right)\;dz.$$

By interchanging summation and integration, we obtain

$$\varphi_{Z}\left(t\right)=\sum_{m=0}^{\infty }\frac{{\left(it\right)}^{m}}{m!}\int_{0}^{1}z^{m}f_{Z}\left(z;\zeta \right)\;dz=\sum_{m=0}^{\infty }\frac{{\left(it\right)}^{m}}{m!}\;\mu_{m}^{\prime }.$$

Finally, substituting the expression of $\mu_{m}^{\prime }$ from Equation (10) yields (13). This completes the proof.    □

**Remark** **4.** *The $m^{th}$ non-central moment of Z can be obtained from the CF $\varphi_{Z}\left(t\right)$ as follows*

$$\mu_{m}^{\prime }={\left.\frac{1}{i^{m}}\frac{d^{m}}{dt^{m}}\varphi_{Z}\left(t\right)\right|}_{t=0},\;m=1,2,\ldots .$$

**Definition** **1** (Cumulant Generating Function)**.** *The cumulant generating function (CGF) of a random variable Z is defined as*

(14) $$K_{Z}\left(t\right)=\log M_{Z}\left(t\right),\;t\in R,$$

*provided that the MGF $M_{Z}\left(t\right)$ exists in a neighborhood of the origin. Substituting from Equation (12) into Equation (14), the CGF of the UASRD is obtained as*

(15) KZ(t)=log∑m=0∞tmm!∑r=0∞2rr4r2π(2r+1)−2mζπ(2r+1)3/2em2ζ22r+1erfcmζ2r+1.

*Consequently, the $m^{\mathrm{th}}$ cumulant $\kappa_{m}$ of Z is obtained by differentiation of the CGF as*

$$\kappa_{m}={\left.\frac{d^{m}}{dt^{m}}K_{Z}\left(t\right)\right|}_{t=0},\;m=1,2,\ldots .$$

*These cumulants facilitate the systematic derivation of key descriptive measures of the UASRD, including $\mu =\kappa_{1}$, $\sigma^{2}=\kappa_{2}$, $\gamma_{1}=\kappa_{3}/\kappa_{2}^{3/2}$, and $\gamma_{2}=\kappa_{4}/\kappa_{2}^{2}$.*

**Theorem** **8** (Incomplete Moments of the UASRD)**.** *Let Z∼UASRD(ζ) with PDF $f_{Z}\left(z;\zeta \right)$ given in Equation (4), where ζ>0. Then, for any real m>−1 and 0<z<1, the $m^{\mathrm{th}}$ incomplete moment of Z up to z, defined by*

$$\psi_{m}\left(z\right)=E\;\left[Z^{m}\;1_{\left\{Z\leq z\right\}}\right]=\int_{0}^{z}t^{m}f_{Z}\left(t;\zeta \right)\;dt,$$

*exists and can be expressed in closed form as follows:*

(16) ψm(z)=∑k=0∞2kk4k2zmπ(2k+1)e−(2k+1)(logz)24ζ2−2mζπ(2k+1)3/2em2ζ22k+1erfc(2k+1)(−logz)+2mζ22ζ2k+1.

**Proof.**  By definition and using Equation (4), the $m^{\mathrm{th}}$ incomplete moment is given by

$$\psi_{m}\left(z\right)=\int_{0}^{z}t^{m}f_{Z}\left(t;\zeta \right)\;dt=\frac{1}{\pi \zeta^{2}}\int_{0}^{z}\frac{-t^{m-1}\log t\;e^{-\frac{\log^{2}\left(t\right)}{4\zeta^{2}}}}{\sqrt{1-e^{-\frac{\log^{2}\left(t\right)}{2\zeta^{2}}}}}\;dt.$$

Applying the transformation y=−logt, so that $t=e^{-y}$ and $dt=-e^{-y}dy$, we have that as *t* increases from 0 to *z*, *y* decreases from ∞ to −logz. Hence, we obtain

$$\psi_{m}\left(z\right)=\frac{1}{\pi \zeta^{2}}\int_{-\log z}^{\infty }\frac{y\;e^{-my}e^{-\frac{y^{2}}{4\zeta^{2}}}}{\sqrt{1-e^{-\frac{y^{2}}{2\zeta^{2}}}}}\;dy.$$

For y>0, let $w=e^{-\frac{y^{2}}{2\zeta^{2}}}\in \left(0,1\right)$. Using the binomial expansion

11−w=∑k=0∞2kk4kwk,|w|<1,weobtain11−e−y22ζ2=∑k=0∞2kk4ke−ky22ζ2.

Since the integrand is nonnegative for m>−1, Tonelli’s theorem justifies the interchange of summation and integration, yielding

ψm(z)=1πζ2∑k=0∞2kk4k∫−logz∞ye−my−(2k+1)y24ζ2dy.

The integral is of the Gaussian type and can be evaluated using the identity

$$\int_{a}^{\infty }y\;e^{-Ay^{2}-By}\;dy=\frac{1}{2A}e^{-Aa^{2}-Ba}-\frac{B\sqrt{\pi }}{4A^{3/2}}e^{\frac{B^{2}}{4A}}\mathrm{erfc}\;\left(\frac{2Aa+B}{2\sqrt{A}}\right),\;A>0.$$

Setting $A=\frac{2k+1}{4\zeta^{2}}$, B=m, and a=−logz, we obtain

$$\begin{array}{cc} \int_{-\log z}^{\infty }y\;e^{-my-\frac{\left(2k+1\right)y^{2}}{4\zeta^{2}}}\;dy & =\frac{2\zeta^{2}}{2k+1}\;z^{m}e^{-\frac{\left(2k+1\right){\left(\log z\right)}^{2}}{4\zeta^{2}}}-\frac{2m\sqrt{\pi }\;\zeta^{3}}{{\left(2k+1\right)}^{3/2}}e^{\frac{m^{2}\zeta^{2}}{2k+1}}\mathrm{erfc}\;\left(\frac{\left(2k+1\right)\left(-\log z\right)+2m\zeta^{2}}{2\zeta \sqrt{2k+1}}\right). \end{array}$$

Substituting this expression into the series representation and simplifying yields Equation (16), which completes the proof.    □

**Remark** **5.** *The incomplete moment $\psi_{m}\left(z\right)$ extends the concept of the standard $m^{\mathrm{th}}$ non-central moment $\mu_{m}^{\prime }$ of the UASRD. Specifically, it reduces to the usual non-central moment when $z\to 1^{-}$, i.e., $\lim_{z\to 1^{-}}\psi_{m}\left(z\right)=\mu_{m}^{\prime }$, which is consistent with Equation (10).*

### 3.4. Mean Residual Life and Mean Inactivity Time

The mean residual life (MRL) and mean inactivity time (MIT) functions are key concepts in reliability theory and survival analysis, quantifying the expected remaining and elapsed lifetimes of a system, respectively. For a random variable Z∼UASRD(ζ) supported on (0,1), the MRL function at z∈(0,1) is defined by

(17) $${\hat{m}}_{r}\left(z;\zeta \right)=E\left[Z-z|Z>z\right]=\frac{1}{R_{Z}\left(z;\zeta \right)}\int_{z}^{1}t\;f_{Z}\left(t;\zeta \right)\;dt-z.$$

Substituting the PDF of the UASRD from Equation (4) into Equation (17), we obtain

(18) $${\hat{m}}_{r}\left(z;\zeta \right)=\frac{1}{\pi \zeta^{2}R_{Z}\left(z;\zeta \right)}\int_{z}^{1}\frac{-\log t\;e^{-\frac{\log^{2}\left(t\right)}{4\zeta^{2}}}}{\sqrt{1-e^{-\frac{\log^{2}\left(t\right)}{2\zeta^{2}}}}}\;dt-z.$$

To evaluate the integral, employ the transformation y=−logt, so that $t=e^{-y}$ and $dt=-e^{-y}dy$. As *t* increases from *z* to 1, *y* decreases from −logz to 0, and Equation (18) becomes

$${\hat{m}}_{r}\left(z;\zeta \right)=\frac{1}{\pi \zeta^{2}R_{Z}\left(z;\zeta \right)}\int_{0}^{-\log z}\frac{y\;e^{-y}\;e^{-\frac{y^{2}}{4\zeta^{2}}}}{\sqrt{1-e^{-\frac{y^{2}}{2\zeta^{2}}}}}\;dy-z.$$

For y>0, let $w=e^{-\frac{y^{2}}{2\zeta^{2}}}\in \left(0,1\right)$. Using the binomial expansion

11−w=∑k=0∞2kk4kwk,|w|<1,weobtain11−e−y22ζ2=∑k=0∞2kk4ke−ky22ζ2.

Since the integrand is nonnegative, Tonelli’s theorem justifies interchanging summation and integration, yielding

(19) m^r(z;ζ)=1πζ2RZ(z;ζ)∑k=0∞2kk4k∫0−logzye−ye−(2k+1)y24ζ2dy−z.

The integral is of the Gaussian type and can be evaluated using the identity

$$\int_{0}^{A}y\;e^{-y-\beta y^{2}}\;dy=\frac{1}{2\beta }\left(1-e^{-\beta A^{2}-A}\right)+\frac{\sqrt{\pi }}{4\beta^{3/2}}\;e^{\frac{1}{4\beta }}\left[\mathrm{erf}\;\left(\frac{1}{2\sqrt{\beta }}\right)-\mathrm{erf}\;\left(\frac{2\beta A+1}{2\sqrt{\beta }}\right)\right].$$

where A>0 and β>0. Setting $\beta =\frac{2k+1}{4\zeta^{2}}$ and A=−logz, we obtain

(20) $$\begin{array}{cc} \int_{0}^{-\log z}y\;e^{-y}\;e^{-\frac{\left(2k+1\right)y^{2}}{4\zeta^{2}}}\;dy\; & =\frac{2\zeta^{2}}{2k+1}\left(1-z\;e^{-\frac{\left(2k+1\right){\left(\log z\right)}^{2}}{4\zeta^{2}}}\right)+\frac{2\sqrt{\pi }\;\zeta^{3}}{{\left(2k+1\right)}^{3/2}}e^{\frac{\zeta^{2}}{2k+1}}\\ \; & \;\times \left[\mathrm{erf}\;\left(\frac{\zeta }{\sqrt{2k+1}}\right)-\mathrm{erf}\;\left(\frac{\left(2k+1\right)\left(-\log z\right)+2\zeta^{2}}{2\zeta \sqrt{2k+1}}\right)\right]. \end{array}$$

Substituting Equation (20) into Equation (19), we obtain

(21) m^rz;ζ=1RZz;ζ∑k=0∞2kk4k{2π2k+11−ze−2k+1logz24ζ2+2ζπ2k+13/2eζ22k+1×erfζ2k+1−erf2k+1−logz+2ζ22ζ2k+1}−z.

Similarly, the MIT function at z∈(0,1) is defined by

(22) $${\hat{m}}_{i}\left(z;\zeta \right)=E\left[z-Z|Z\leq z\right]=\frac{1}{F_{Z}\left(z;\zeta \right)}\int_{0}^{z}F_{Z}\left(y;\zeta \right)\;dy=z-\frac{1}{F_{Z}\left(z;\zeta \right)}\int_{0}^{z}t\;f_{Z}\left(t;\zeta \right)\;dt.$$

Substituting the PDF of the UASRD from Equation (4) into Equation (22), we obtain

$${\hat{m}}_{i}\left(z;\zeta \right)=z-\frac{1}{\pi \zeta^{2}F_{Z}\left(z;\zeta \right)}\int_{0}^{z}\frac{-t\log t\;e^{-\frac{\log^{2}\left(t\right)}{4\zeta^{2}}}}{\sqrt{1-e^{-\frac{\log^{2}\left(t\right)}{2\zeta^{2}}}}}\;dt.$$

Applying the transformation y=−logt as before, we obtain

$${\hat{m}}_{i}\left(z;\zeta \right)=z-\frac{1}{\pi \zeta^{2}F_{Z}\left(z;\zeta \right)}\int_{-\log z}^{\infty }\frac{y\;e^{-y}\;e^{-\frac{y^{2}}{4\zeta^{2}}}}{\sqrt{1-e^{-\frac{y^{2}}{2\zeta^{2}}}}}\;dy.$$

Using the same binomial expansion and integration technique as in the derivation of the MRL function, a series representation of the MIT function can be obtained. Specifically, we have

(23) m^i(z;ζ)=z−1πζ2FZ(z;ζ)∑k=0∞2kk4k∫−logz∞ye−ye−(2k+1)y24ζ2dy.

The integral is of the Gaussian type and can be evaluated using the identity

$$\int_{A}^{\infty }y\;e^{-y-\beta y^{2}}\;dy=\frac{e^{-A(A\beta +1)}}{2\beta }-\frac{\sqrt{\pi }}{4\beta^{3/2}}\;e^{A^{2}\beta -A\left(A\beta +1\right)+A+\frac{1}{4\beta }}\mathrm{erfc}\left(\frac{2A\beta +1}{2\sqrt{\beta }}\right).$$

where A>0 and β>0. Setting $\beta =\frac{2k+1}{4\zeta^{2}}$ and A=−logz, we obtain

(24) $$\begin{array}{cc} \int_{-\log z}^{\infty }y\;e^{-y}\;e^{-\frac{\left(2k+1\right)y^{2}}{4\zeta^{2}}}\;dy\; & =\frac{2\zeta^{2}}{{\left(2k+1\right)}^{2}}e^{-\frac{\left(2k+1\right){\left(\log z\right)}^{2}}{4\zeta^{2}}}\{-\zeta \sqrt{\pi (2k+1)}\;e^{\frac{\zeta^{2}}{2k+1}+\frac{\left(2k+1\right)\log^{2}\left(z\right)}{4\zeta^{2}}}\\ \; & \;\times \mathrm{erfc}\left[\frac{\zeta }{\sqrt{2k+1}}\left(1-\frac{\left(2k+1)\log(z\right)}{2\zeta^{2}}\right)\right]+\left(2k+1\right)z\}. \end{array}$$

Substituting Equation (24) into Equation (23), we obtain

(25) m^i(z;ζ)=z−1FZ(z;ζ)∑k=0∞2kk4k2π(2k+1)2e−(2k+1)(logz)24ζ2{−ζπ(2k+1)eζ22k+1+(2k+1)log2(z)4ζ2×erfcζ2k+11−(2k+1)log(z)2ζ2+(2k+1)z}.

Equations (21) and (25) provide explicit series representations for the MRL and MIT functions of the UASRD, respectively. These expressions facilitate stable numerical evaluation of reliability characteristics across z∈(0,1) and ζ>0.

### 3.5. Order Statistics

Order statistics play a crucial role in many areas of statistical theory and applications, particularly in survival analysis and quality control. In this subsection, we present the order statistics of the proposed UASRD. Let $Z_{1:n}\leq Z_{2:n}\leq \ldots \leq Z_{n:n}$ denote the order statistics of a random sample of size *n* drawn from Z∼UASRD(ζ) supported on (0,1). The PDF of the $r^{th}$ order statistic at z∈(0,1), r=1,2,…,n, is given by

$$f_{Z_{r:n}}\left(z;\zeta \right)=\frac{n!}{(r-1)!(n-r)!}\;{\left[F_{Z}\left(z;\zeta \right)\right]}^{r-1}\;{\left[1-F_{Z}\left(z;\zeta \right)\right]}^{n-r}\;f_{Z}\left(z;\zeta \right),$$

and its corresponding CDF is given by

FZr:n(z;ζ)=∑k=rn∑m=0n−knkn−km(−1)m[FZ(z;ζ)]m+k.

Substituting the CDF and PDF of the UASRD from Equations (3) and (4), respectively, yields

(26) $$\begin{array}{cc} f_{Z_{r:n}}\left(z;\zeta \right)\; & =\frac{n!}{(r-1)!(n-r)!}{\left[1-\frac{2}{\pi }\;\arcsin\;\left(\sqrt{1-e^{-\frac{\log^{2}\left(z\right)}{2\zeta^{2}}}}\right)\right]}^{r-1}\\ \; & \;\times {\left[\frac{2}{\pi }\;\arcsin\;\left(\sqrt{1-e^{-\frac{\log^{2}\left(z\right)}{2\zeta^{2}}}}\right)\right]}^{n-r}\left(\frac{-\log z\;e^{-\frac{\log^{2}\left(z\right)}{4\zeta^{2}}}}{\pi \zeta^{2}z\sqrt{1-e^{-\frac{\log^{2}\left(z\right)}{2\zeta^{2}}}}}\right), \end{array}$$

and

(27) FZr:n(z;ζ)=∑k=rn∑m=0n−knkn−km(−1)m1−2πarcsin1−e−log2(z)2ζ2m+k.

In particular, the first (minimum) and last (maximum) order statistics correspond to r=1 and r=n, respectively. Their PDFs are given explicitly as follows:

- **Minimum:** $Z_{1:n}$
  $$f_{Z_{1:n}}\left(z;\zeta \right)=n{\left[\frac{2}{\pi }\arcsin\;\left(\sqrt{1-e^{-\frac{\log^{2}\left(z\right)}{2\zeta^{2}}}}\right)\right]}^{\;n-1}\left(\frac{-\log z\;e^{-\frac{\log^{2}\left(z\right)}{4\zeta^{2}}}}{\pi \zeta^{2}z\sqrt{1-e^{-\frac{\log^{2}\left(z\right)}{2\zeta^{2}}}}}\right).$$
- **Maximum:** $Z_{n:n}$
  $$f_{Z_{n:n}}\left(z;\zeta \right)=n{\left[1-\frac{2}{\pi }\;\arcsin\;\left(\sqrt{1-e^{-\frac{\log^{2}\left(z\right)}{2\zeta^{2}}}}\right)\right]}^{\;n-1}\left(\frac{-\log z\;e^{-\frac{\log^{2}\left(z\right)}{4\zeta^{2}}}}{\pi \zeta^{2}z\sqrt{1-e^{-\frac{\log^{2}\left(z\right)}{2\zeta^{2}}}}}\right).$$

### 3.6. Stochastic Ordering Properties

To further understand the comparative behavior of the proposed UASRD model, we investigate its stochastic ordering properties. Let $Z_{1}∼\mathrm{UASRD}\left(\zeta_{1}\right)$ and $Z_{2}∼\mathrm{UASRD}\left(\zeta_{2}\right)$ with $\zeta_{1}<\zeta_{2}$ and common support (0,1). The stochastic comparison of $Z_{1}$ and $Z_{2}$ provides insight into reliability characteristics and risk behavior as the shape parameter varies. In particular, we consider the likelihood ratio order (LRO) and the hazard rate order (HRO).

#### 3.6.1. Likelihood Ratio Order

The random variable $Z_{1}$ is said to be smaller than $Z_{2}$ in the LRO, denoted $Z_{1}\leq_{LRO}Z_{2}$, if the ratio $r_{1}=\frac{f_{Z_{1}}\left(z;\zeta_{1}\right)}{f_{Z_{2}}\left(z;\zeta_{2}\right)}$ is non-increasing in z∈(0,1). Using the PDF of the UASRD given in Equation (4), we obtain

$$\begin{array}{c} r_{1}={\left(\frac{\zeta_{2}}{\zeta_{1}}\right)}^{2}\;e^{\frac{\log^{2}\left(z\right)}{4}\left(\frac{1}{\zeta_{2}^{2}}-\frac{1}{\zeta_{1}^{2}}\right)}\sqrt{\frac{1-e^{-\frac{\log^{2}\left(z\right)}{2\zeta_{2}^{2}}}}{1-e^{-\frac{\log^{2}\left(z\right)}{2\zeta_{1}^{2}}}}}. \end{array}$$

Let x=−logz>0, so that $\log^{2}\left(z\right)=x^{2}$. Then the ratio becomes

(28) $$r_{1}={\left(\frac{\zeta_{2}}{\zeta_{1}}\right)}^{2}\;e^{\frac{x^{2}}{4}\left(\frac{1}{\zeta_{2}^{2}}-\frac{1}{\zeta_{1}^{2}}\right)}\sqrt{\frac{1-e^{-\frac{x^{2}}{2\zeta_{2}^{2}}}}{1-e^{-\frac{x^{2}}{2\zeta_{1}^{2}}}}}.$$

Since $\zeta_{1}<\zeta_{2}$, we have $\frac{1}{\zeta_{2}^{2}}-\frac{1}{\zeta_{1}^{2}}<0$ and therefore the exponential term is strictly decreasing in *x*. Moreover, consider the function $g_{\zeta }\left(x\right)=1-e^{-\frac{x^{2}}{2\zeta^{2}}}$, x>0. For $\zeta_{1}<\zeta_{2}$, it follows that $g_{\zeta_{2}}\left(x\right)<g_{\zeta_{1}}\left(x\right),$ and hence $\frac{g_{\zeta_{2}}\left(x\right)}{g_{\zeta_{1}}\left(x\right)}$ is decreasing in x>0. Consequently, the square-root term is also decreasing in *x*. Since both components are decreasing in *x*, the product $r_{1}$ is decreasing in *x*, and therefore $r_{1}$ is non-increasing in z∈(0,1). Thus, we conclude that $Z_{1}\leq_{LRO}Z_{2}$ whenever $\zeta_{1}<\zeta_{2}$.

#### 3.6.2. Hazard Rate Order

The HRO is defined by $Z_{1}\leq_{HRO}Z_{2}$ if and only if $h_{Z_{1}}\left(z;\zeta_{1}\right)\geq h_{Z_{2}}\left(z;\zeta_{2}\right)$ for all z∈(0,1), where $h_{Z}\left(z;\zeta \right)$ is given in Equation (6). From this, we obtain

$$r_{2}=\frac{h_{Z_{1}}\left(z;\zeta_{1}\right)}{h_{Z_{2}}\left(z;\zeta_{2}\right)}={\left(\frac{\zeta_{2}}{\zeta_{1}}\right)}^{2}\;e^{\frac{\log^{2}\left(z\right)}{4}\left(\frac{1}{\zeta_{2}^{2}}-\frac{1}{\zeta_{1}^{2}}\right)}\;\frac{\arcsin\;\left(\sqrt{1-e^{-\frac{\log^{2}\left(z\right)}{2\zeta_{2}^{2}}}}\right)}{\arcsin\;\left(\sqrt{1-e^{-\frac{\log^{2}\left(z\right)}{2\zeta_{1}^{2}}}}\right)}.$$

Again, let x=−logz>0. Then, we have

(29) $$r_{2}={\left(\frac{\zeta_{2}}{\zeta_{1}}\right)}^{2}\;e^{\frac{x^{2}}{4}\left(\frac{1}{\zeta_{2}^{2}}-\frac{1}{\zeta_{1}^{2}}\right)}\frac{\arcsin\;\left(\sqrt{1-e^{-\frac{x^{2}}{2\zeta_{2}^{2}}}}\right)}{\arcsin\;\left(\sqrt{1-e^{-\frac{x^{2}}{2\zeta_{1}^{2}}}}\right)}.$$

The overall monotonic behavior depends on the interaction between the exponential and the arcsine ratio. For small *x* (i.e., z→1), the exponential term dominates and tends to favor $h_{Z_{1}}\left(z;\zeta_{1}\right)<h_{Z_{2}}\left(z;\zeta_{2}\right)$. For large *x* (i.e., z→0), the arcsine ratio may dominate, potentially reversing the inequality. Therefore, unlike the likelihood ratio order, the hazard rate order is not globally preserved for all parameter values without additional restrictions. The ordering may depend on the region of the support and specific choices of $\zeta_{1}$ and $\zeta_{2}$.

### 3.7. Entropy Measures

Entropy provides a quantitative measure of uncertainty and information content associated with a probability distribution and plays a central role in information theory, reliability analysis, and risk assessment. For the UASRD, several generalized entropy measures can be derived in closed form by evaluating a common integral involving the PDF. Let Z∼UASRD(ζ) with PDF given in Equation (4). For δ>0, δ≠1, consider the integral

(30) $$I_{\delta }\left(\zeta \right)=\int_{0}^{1}{\left[f_{Z}\left(z;\zeta \right)\right]}^{\delta }dz.$$

Substituting the PDF from Equation (4) and simplifying, we obtain

$$I_{\delta }\left(\zeta \right)=\frac{1}{\pi^{\delta }\zeta^{2\delta }}\int_{0}^{1}\frac{{\left(-\log z\right)}^{\delta }\;z^{-\delta }e^{-\frac{\delta \log^{2}\left(z\right)}{4\zeta^{2}}}}{{\left(1-e^{-\frac{\log^{2}\left(z\right)}{2\zeta^{2}}}\right)}^{\frac{\delta }{2}}}\;dz.$$

Applying the transformation y=−logz, so that $z=e^{-y}$ and $dz=-e^{-y}dy$, yields

$$I_{\delta }\left(\zeta \right)=\frac{1}{\pi^{\delta }\zeta^{2\delta }}\int_{0}^{\infty }\frac{y^{\delta }e^{-(1-\delta )y}e^{-\frac{\delta y^{2}}{4\zeta^{2}}}}{{\left(1-e^{-\frac{y^{2}}{2\zeta^{2}}}\right)}^{\delta /2}}\;dy.$$

For y>0, define $w=e^{-\frac{y^{2}}{2\zeta^{2}}}\in \left(0,1\right)$, such that |w|<1. Using the binomial expansion

(1−w)−δ2=∑k=0∞δ2+k−1kwk,weobtain11−e−y22ζ2δ2=∑k=0∞δ2+k−1ke−ky22ζ2.

Since the integrand is nonnegative for δ>0, Tonelli’s theorem justifies the interchange of summation and integration, leading to

Iδ(ζ)=1πδζ2δ∑k=0∞δ2+k−1k∫0∞yδe−(1−δ)ye−(2k+δ)y24ζ2dy.

The integral is of the Gaussian type and can be evaluated using the identity

∫0∞yδeAy−βy2dy=12β−δ2−1{AΓδ2+1F11δ2+1;32;A24β+βΓδ+12×1F1δ+12;12;A24β},β>0,

where F11(a;b;z) denotes the Kummer confluent hypergeometric function. Setting $\beta =\frac{2k+\delta }{4\zeta^{2}}$ and A=1−δ, we obtain

(31) Iδ(ζ)=1πδζ2δ∑k=0∞δ2+k−1k2δζδ+1(δ+2k)−δ2−1{(δ−1)δζΓδ2×1F1δ+22;32;(δ−1)2ζ22k+δ+Γδ+12δ+2kF11δ+12;12;(δ−1)2ζ22k+δ}.

Equation (31) provides a general expression for the integral $I_{\delta }\left(\zeta \right)$, which forms the basis for several entropy measures associated with the UASRD.

#### 3.7.1. Rényi Entropy

The Rényi (R) entropy [16] of order δ>0, δ≠1 is defined by

(32) Rδ(ζ)=11−δlogIδ(ζ)=11−δlog{1πδζ2δ∑k=0∞δ2+k−1k2δζδ+1(δ+2k)−δ2−1×[(δ−1)δζΓδ2F11δ+22;32;(δ−1)2ζ22k+δ+Γδ+12×δ+2kF11δ+12;12;(δ−1)2ζ22k+δ]}.

#### 3.7.2. Arimoto Entropy

The Arimoto (A) entropy [17] of order δ>0, δ≠1 is defined by

(33) Aδ(ζ)=δ1−δIδ(ζ)1δ−1=δ1−δ{(1πδζ2δ∑k=0∞δ2+k−1k2δζδ+1(δ+2k)−δ2−1×[(δ−1)δζΓδ2F11δ+22;32;(δ−1)2ζ22k+δ+Γδ+12δ+2kF11δ+12;12;(δ−1)2ζ22k+δ])1δ−1}.

#### 3.7.3. Tsallis Entropy

The Tsallis (T) entropy [18,19] of order δ>0, δ≠1, is defined by

(34) Tδ(ζ)=1δ−11−Iδ(ζ)=1δ−1{1−(1πδζ2δ∑k=0∞δ2+k−1k2δζδ+1(δ+2k)−δ2−1×[(δ−1)δζΓδ2F11δ+22;32;(δ−1)2ζ22k+δ+Γδ+12δ+2kF11δ+12;12;(δ−1)2ζ22k+δ])}.

#### 3.7.4. Havrda–Charvát Entropy

The Havrda–Charvát (HC) entropy [20] of order δ>0, δ≠1, is defined by

(35) HCδ(ζ)=121−δ−1Iδ(ζ)−1=121−δ−1{(1πδζ2δ∑k=0∞δ2+k−1k2δζδ+1(δ+2k)−δ2−1×[(δ−1)δζΓδ2F11δ+22;32;(δ−1)2ζ22k+δ+Γδ+12δ+2kF11δ+12;12;(δ−1)2ζ22k+δ])−1}.

#### 3.7.5. Mathai–Haubold Entropy

The Mathai–Haubold (MH) entropy [21] of order δ>0, δ≠1, is defined by

(36) MHδ(ζ)=1δ−1I(2−δ)(ζ)−1=1δ−1{(1π2−δζ4−2δ∑k=0∞k−δ2k22−δζ3−δ×(2k+2−δ)δ2−2[(1−δ)(2−δ)ζΓ1−δ2×1F12−δ2;32;(1−δ)2ζ22k+2−δ+Γ3−δ22k+2−δ×1F13−δ2;12;(1−δ)2ζ22k+2−δ])−1}.

#### 3.7.6. Shannon Entropy

Shannon ($S_{h}$) entropy [22] is a fundamental measure of uncertainty for continuous random variables. For a random variable *Z* with PDF $f_{Z}\left(z;\zeta \right)$, it is defined by

$$S_{h}\left(\zeta \right)=-E\;\left[\log f_{Z}\left(Z;\zeta \right)\right].$$

Let Z∼UASRD(ζ) with PDF given in Equation (4). Substituting $f_{Z}\left(z;\zeta \right)$ into the definition yields

(37) $$\begin{array}{cc} S_{h}\left(\zeta \right)=-E\;\left[\log\left(-\frac{\log Z\;e^{-\frac{\log^{2}\left(Z\right)}{4\zeta^{2}}}}{\pi \zeta^{2}Z\sqrt{1-e^{-\frac{\log^{2}\left(Z\right)}{2\zeta^{2}}}}}\right)\right]\; & =\log\left(\pi \zeta^{2}\right)+E\left[\log Z\right]-E\left[\log\left(-\log Z\right)\right]\\ \; & \;+\frac{1}{4\zeta^{2}}E\left[\log^{2}Z\right]+\frac{1}{2}E\;\left[\log\;\left(1-e^{-\frac{\log^{2}Z}{2\zeta^{2}}}\right)\right]. \end{array}$$

Let X=−logZ. Since $Z=e^{-X}$ and X∼ASRD(ζ) with PDF given in Equation (2), it follows that

(38) $$S_{h}\left(\zeta \right)=\log\left(\pi \zeta^{2}\right)-E\left[X\right]-E\left[\log X\right]+\frac{1}{4\zeta^{2}}E\left[X^{2}\right]+\frac{1}{2}E\;\left[\log\;\left(1-e^{-\frac{X^{2}}{2\zeta^{2}}}\right)\right].$$

To simplify the last two expectations, let $Y=e^{-\frac{X^{2}}{2\zeta^{2}}}\in \left(0,1\right).$ A direct change of variables from the ASRD density shows that

$$f_{Y}\left(y\right)=\frac{1}{\pi \sqrt{y(1-y)}},\;0<y<1,$$

that is, $Y∼\mathrm{Beta}\;\left(\frac{1}{2},\frac{1}{2}\right)$. Consequently,

$$E\left[X^{2}\right]=-2\zeta^{2}\;E\left[\log Y\right]\;\mathrm{and}\;E\;\left[\log\;\left(1-e^{-\frac{X^{2}}{2\zeta^{2}}}\right)\right]=E\left[\log\left(1-Y\right)\right].$$

Using the well-known expectations for the Beta $\left(\frac{1}{2},\frac{1}{2}\right)$ distribution,

E[logY]=E[log(1−Y)]=−2log2,

we obtain

$$\frac{1}{4\zeta^{2}}E\left[X^{2}\right]=\log2\;\mathrm{and}\;\frac{1}{2}E\;\left[\log\;\left(1-e^{-\frac{X^{2}}{2\zeta^{2}}}\right)\right]=-\log2.$$

Hence, these two contributions cancel exactly. Therefore,

(39) $$S_{h}\left(\zeta \right)=\log\left(\pi \zeta^{2}\right)-E\left[X\right]-E\left[\log X\right],\;X∼\mathrm{ASRD}\left(\zeta \right).$$

We now evaluate E[X]. By definition,

$$E\left[X\right]=\int_{0}^{\infty }\frac{e^{-x^{2}/\left(4\zeta^{2}\right)}\;x^{2}}{\pi \zeta^{2}\sqrt{1-e^{-x^{2}/\left(2\zeta^{2}\right)}}}\;dx\;\mathrm{and}\;E\left[\log X\right]=\int_{0}^{\infty }\frac{xe^{-\frac{x^{2}}{4\zeta^{2}}}\log x}{\pi \zeta^{2}\sqrt{1-e^{-\frac{x^{2}}{2\zeta^{2}}}}}\;dx.$$

Let $u=\frac{x^{2}}{2\zeta^{2}}$, so that $x=\zeta \sqrt{2u}$ and $dx=\frac{\zeta }{\sqrt{2u}}\;du$. Then, we obtain

$$E\left[X\right]=\frac{\sqrt{2}\;\zeta }{\pi }\int_{0}^{\infty }u^{1/2}\;e^{-u/2}\;{\left(1-e^{-u}\right)}^{-1/2}\;du.$$

Using the binomial expansion 11−e−u=∑k=0∞2kk4ke−ku, we obtain

E[X]=2ζπ∑k=0∞2kk4k∫0∞u1/2e−(k+12)udu.

Using Euler’s Gamma integral $\int_{0}^{\infty }u^{r-1}e^{-au}du=\Gamma \left(r\right)a^{-r}$, we have

$$\int_{0}^{\infty }u^{1/2}e^{-(k+\frac{1}{2})u}\;du=\frac{\Gamma (\frac{3}{2})}{{\left(k+\frac{1}{2}\right)}^{3/2}}=\frac{\sqrt{\pi }}{2}\frac{1}{{\left(k+\frac{1}{2}\right)}^{3/2}}.$$

Therefore, we obtain

(40) E[X]=ζ2π∑k=0∞2kk4k(k+12)3/2.

Similarly, proceeding as above, we obtain

(41) E[logX]=logζ+12π∫0∞e−u/21−e−ulog(2u)du=logζ+12π∑k=0∞2kk4k∫0∞e−(k+12)ulog(2u)du=logζ+12π∑k=0∞2kk4klog2−γ−log(k+12)k+12,

where γ denotes Euler’s constant. Substituting (40) and (41) into (39), the Shannon entropy of the UASRD is given by

(42) Sh(ζ)=log(πζ)−ζ2π∑k=0∞2kk4k(k+12)3/2−12π∑k=0∞2kk4klog2−γ−log(k+12)k+12.

### 3.8. Stress–Strength Reliability

Stress–strength reliability (SSR) is defined as the probability that a system’s strength exceeds the applied stress. Let $Z_{1}$ and $Z_{2}$ represent the system strength and applied stress, respectively, where $Z_{1}∼\mathrm{UASRD}\left(\zeta_{1}\right)$ and $Z_{2}∼\mathrm{UASRD}\left(\zeta_{2}\right)$ with parameters $\zeta_{1},\zeta_{2}>0$. Assume that $Z_{1}$ and $Z_{2}$ are independent random variables sharing the common support (0,1). The system operates successfully if $Z_{1}>Z_{2}$, and the SSR is therefore given by

(43) $$R_{ss}=P\left(Z_{1}>Z_{2}\right)=\int_{0}^{1}f_{Z_{1}}\left(z;\zeta_{1}\right)\;F_{Z_{2}}\left(z;\zeta_{2}\right)\;dz.$$

Substituting the PDF and CDF of the UASRD from Equations (3) and (4) into Equation (43) yields

(44) $$\begin{array}{cc} R_{ss}\; & =\int_{0}^{1}\left(-\frac{\log z\;e^{-\frac{\log^{2}\left(z\right)}{4\zeta_{1}^{2}}}}{\pi \zeta_{1}^{2}z\sqrt{1-e^{-\frac{\log^{2}\left(z\right)}{2\zeta_{1}^{2}}}}}\right)\left[1-\frac{2}{\pi }\arcsin\;\left(\sqrt{1-e^{-\frac{\log^{2}\left(z\right)}{2\zeta_{2}^{2}}}}\right)\right]dz. \end{array}$$

To evaluate Equation (44), consider the transformation x=−logz, so that $z=e^{-x}$, $dz=-e^{-x}dx$, and x∈(0,∞). After simplification, we obtain

(45) $$\begin{array}{cc} R_{ss}\; & =\int_{0}^{\infty }\frac{x\;e^{-\frac{x^{2}}{4\zeta_{1}^{2}}}}{\pi \zeta_{1}^{2}\sqrt{1-e^{-\frac{x^{2}}{2\zeta_{1}^{2}}}}}\left[1-\frac{2}{\pi }\arcsin\;\left(\sqrt{1-e^{-\frac{x^{2}}{2\zeta_{2}^{2}}}}\right)\right]dx. \end{array}$$

Next, introduce the transformation $w=e^{-\frac{x^{2}}{2\zeta_{1}^{2}}}$, 0<w<1, so that $dw=-\frac{x}{\zeta_{1}^{2}}w\;dx$ and $e^{-\frac{x^{2}}{2\zeta_{2}^{2}}}=w^{\;\alpha }$, where $\alpha ={\left(\frac{\zeta_{1}}{\zeta_{2}}\right)}^{2}>0$. Substituting into Equation (45) gives

(46) $$\begin{array}{cc} R_{ss}\; & =\frac{1}{\pi }\int_{0}^{1}\frac{1-\frac{2}{\pi }\arcsin\;\left(\sqrt{1-w^{\alpha }}\right)}{\sqrt{w(1-w)}}\;dw. \end{array}$$

Now, we set $w=\sin^{2}\theta$, $\theta \in (0,\frac{\pi }{2})$. Then dw=2sinθcosθdθ and $\sqrt{w(1-w)}=\sin\theta \cos\theta$, so that $\frac{dw}{\sqrt{w(1-w)}}=2\;d\theta$. Consequently, we obtain

(47) $$\begin{array}{cc} R_{ss}\; & =\frac{2}{\pi }\int_{0}^{\frac{\pi }{2}}\left[1-\frac{2}{\pi }\arcsin\;\left(\sqrt{1-\sin^{2\alpha }\theta }\right)\right]d\theta . \end{array}$$

Using the identity $\arcsin\left(\sqrt{1-u^{2}}\right)=\arccos\left(u\right)=\frac{\pi }{2}-\arcsin\left(u\right)$ for u∈[0,1], we obtain

$$\arcsin\;\left(\sqrt{1-\sin^{2\alpha }\theta }\right)=\frac{\pi }{2}-\arcsin\;\left(\sin^{\alpha }\theta \right).$$

Substitution into Equation (47) yields the compact representation

(48) $$R_{ss}=\frac{4}{\pi^{2}}\int_{0}^{\frac{\pi }{2}}\arcsin\;\left(\sin^{\alpha }\theta \right)\;d\theta ,\;\alpha ={\left(\frac{\zeta_{1}}{\zeta_{2}}\right)}^{2}.$$

In the particular case $\zeta_{1}=\zeta_{2}$ (i.e., α=1), arcsin(sinθ)=θ on $\left[0,\frac{\pi }{2}\right]$, and therefore

$$R_{ss}=\frac{4}{\pi^{2}}\int_{0}^{\frac{\pi }{2}}\theta \;d\theta =\frac{1}{2},$$

which agrees with the symmetry property. Furthermore, using the power-series expansion

$$\arcsin\left(t\right)=\sum_{k=0}^{\infty }\frac{(2k)!}{4^{k}{\left(k!\right)}^{2}\left(2k+1\right)}\;t^{2k+1},\;\left|t\right|\leq 1,$$

and noting that $0\leq \sin^{\alpha }\theta \leq 1$ on $\left[0,\frac{\pi }{2}\right]$, the interchange of summation and integration is justified by uniform convergence. Hence,

(49) $$\begin{array}{cc} R_{ss}\; & =\frac{4}{\pi^{2}}\sum_{k=0}^{\infty }\frac{(2k)!}{4^{k}{\left(k!\right)}^{2}\left(2k+1\right)}\int_{0}^{\frac{\pi }{2}}\sin^{\alpha (2k+1)}\theta \;d\theta \\ \; & =\frac{2}{\pi^{2}}\sum_{k=0}^{\infty }\frac{(2k)!}{4^{k}{\left(k!\right)}^{2}\left(2k+1\right)}\;B\;\left(\frac{\alpha (2k+1)+1}{2},\frac{1}{2}\right), \end{array}$$

where B(·,·) denotes the Beta function. Equivalently, using $B\left(a,\frac{1}{2}\right)=\frac{\Gamma \left(a\right)\Gamma \left(\frac{1}{2}\right)}{\Gamma (a+\frac{1}{2})}$ and $\Gamma \left(\frac{1}{2}\right)=\sqrt{\pi }$, we obtain

(50) $$R_{ss}=\frac{2}{\pi^{3/2}}\sum_{k=0}^{\infty }\frac{(2k)!}{4^{k}{\left(k!\right)}^{2}\left(2k+1\right)}\frac{\Gamma \;\left(\frac{\alpha (2k+1)+1}{2}\right)}{\Gamma \;\left(\frac{\alpha (2k+1)+2}{2}\right)},\;\alpha ={\left(\frac{\zeta_{1}}{\zeta_{2}}\right)}^{2}.$$

## 4. Non-Bayesian Inference

This section addresses the estimation of the parameter ζ for the UASRD, as well as the associated functions $R_{Z}\left(z;\zeta \right)$ and $h_{Z}\left(z;\zeta \right)$, using classical (non-Bayesian) inference methods. The classical methods are categorized into likelihood-based and spacing-based techniques. Asymptotic confidence intervals (ACIs) are also provided.

### 4.1. Maximum Likelihood Estimation

Let $z_{1},z_{2},\ldots ,z_{n}$ be a random sample of size *n* drawn from the UASRD with parameter ζ>0 and PDF $f_{Z}\left(z;\zeta \right)$ given in Equation (4). The likelihood function corresponding to this sample is

(51) $$L\left(\zeta \right)=\prod_{i=1}^{n}f_{Z}\left(z_{i};\zeta \right)=\prod_{i=1}^{n}\left(-\frac{\log z_{i}\;e^{-\frac{\log^{2}\left(z_{i}\right)}{4\zeta^{2}}}}{\pi \zeta^{2}z_{i}\sqrt{1-e^{-\frac{\log^{2}\left(z_{i}\right)}{2\zeta^{2}}}}}\right),\;0<z_{i}<1.$$

Taking the natural logarithm of L(ζ) yields the log-likelihood function as

(52) $$\begin{array}{cc} \ell_{\mathrm{ML}}\left(\zeta \right)=\sum_{i=1}^{n}\log f_{Z}\left(z_{i};\zeta \right)\; & =-n\log\pi -2n\log\zeta -\sum_{i=1}^{n}\log z_{i}+\sum_{i=1}^{n}\log\left(-\log z_{i}\right)\\ \; & \;-\frac{1}{4\zeta^{2}}\sum_{i=1}^{n}\log^{2}z_{i}-\frac{1}{2}\sum_{i=1}^{n}\log\;\left(1-e^{-\frac{\log^{2}\left(z_{i}\right)}{2\zeta^{2}}}\right). \end{array}$$

Then, the maximum likelihood (ML) estimator of the parameter ζ, denoted by ${\hat{\zeta }}_{\mathrm{ML}}$, is obtained by maximizing $\ell_{\mathrm{ML}}\left(\zeta \right)$ in Equation (52). Specifically, ${\hat{\zeta }}_{\mathrm{ML}}=\arg\max_{\zeta >0}\ell_{\mathrm{ML}}\left(\zeta \right)$. Differentiating $\ell_{\mathrm{ML}}\left(\zeta \right)$ with respect to ζ and setting it equal to zero leads to the score equation:

(53) $$\frac{\partial \ell_{\mathrm{ML}}\left(\zeta \right)}{\partial \zeta }=-\frac{2n}{\zeta }+\frac{1}{2\zeta^{3}}\sum_{i=1}^{n}\log^{2}z_{i}+\frac{1}{2\zeta^{3}}\sum_{i=1}^{n}\left(\frac{\log^{2}z_{i}\;e^{-\frac{\log^{2}\left(z_{i}\right)}{2\zeta^{2}}}}{1-e^{-\frac{\log^{2}\left(z_{i}\right)}{2\zeta^{2}}}}\right)=0.$$

### 4.2. Maximum Product of Spacings Estimation

The maximum product of spacings (MPS) method provides a robust alternative for parameter estimation by maximizing the geometric mean of the spacings or, equivalently, the sum of their logarithms [23]. Let $z_{1:n}<z_{2:n}<\ldots <z_{n:n}$ denote an ordered sample of size *n* drawn from the UASRD with parameter ζ>0 and CDF $F_{Z}\left(\cdot ;\zeta \right)$ given in Equation (3). The product of the spacing function corresponding to this sample is

(54) $$P\left(\zeta \right)={\left\{\prod_{i=1}^{n+1}D_{i}\left(\zeta \right)\right\}}^{\frac{1}{n+1}},\;D_{i}\left(\zeta \right)=\left\{\begin{array}{cc} F_{Z}\left(z_{1:n};\zeta \right), & i=1,\\ F_{Z}\left(z_{i:n};\zeta \right)-F_{Z}\left(z_{(i-1):n};\zeta \right), & i=2,\ldots ,n,\\ 1-F_{Z}\left(z_{n:n};\zeta \right), & i=n+1, \end{array}\right.$$

where $D_{i}\left(\zeta \right)$ represent the spacings and satisfy $\sum_{i=1}^{n+1}D_{i}\left(\zeta \right)=1$. Explicitly, we have

(55) $$\begin{array}{cc} P(\zeta )= & \{\left[1-\frac{2}{\pi }\arcsin\;\left(\sqrt{1-e^{-\frac{\log^{2}\left(z_{1:n}\right)}{2\zeta^{2}}}}\right)\right]\times \left[\frac{2}{\pi }\arcsin\;\left(\sqrt{1-e^{-\frac{\log^{2}\left(z_{n:n}\right)}{2\zeta^{2}}}}\right)\right]\\ & \times \prod_{i=2}^{n}\frac{2}{\pi }\left[\arcsin\;\left(\sqrt{1-e^{-\frac{\log^{2}\left(z_{(i-1):n}\right)}{2\zeta^{2}}}}\right)-\arcsin\;\left(\sqrt{1-e^{-\frac{\log^{2}\left(z_{i:n}\right)}{2\zeta^{2}}}}\right)\right]{\}}^{\frac{1}{n+1}}. \end{array}$$

Accordingly, taking the natural logarithm of P(ζ) yields the log product of the spacings function as

(56) $$\begin{array}{cc} \ell_{\mathrm{MPS}}\left(\zeta \right)\; & =\frac{1}{n+1}\{\left(n-1\right)\log\frac{2}{\pi }+\log\left[1-\frac{2}{\pi }\arcsin\;\left(\sqrt{1-e^{-\frac{\log^{2}\left(z_{1:n}\right)}{2\zeta^{2}}}}\right)\right]\\ \; & \;+\log\left[\frac{2}{\pi }\arcsin\;\left(\sqrt{1-e^{-\frac{\log^{2}\left(z_{n:n}\right)}{2\zeta^{2}}}}\right)\right]+\sum_{i=2}^{n}\log[\arcsin\;\left(\sqrt{1-e^{-\frac{\log^{2}\left(z_{(i-1):n}\right)}{2\zeta^{2}}}}\right)\\ \; & \;-\arcsin\;\left(\sqrt{1-e^{-\frac{\log^{2}\left(z_{i:n}\right)}{2\zeta^{2}}}}\right)]\}. \end{array}$$

Then, the MPS estimator of the parameter ζ, denoted by ${\hat{\zeta }}_{\mathrm{MPS}}$, is obtained by maximizing $\ell_{\mathrm{MPS}}\left(\zeta \right)$ in Equation (56). Specifically, ${\hat{\zeta }}_{\mathrm{MPS}}=\arg\max_{\zeta >0}\ell_{\mathrm{MPS}}\left(\zeta \right)$. Differentiating $\ell_{\mathrm{MPS}}\left(\zeta \right)$ with respect to ζ and setting it equal to zero leads to the score equation:

(57) $$\begin{array}{cc} \frac{\partial \ell_{\mathrm{MPS}}\left(\zeta \right)}{\partial \zeta }=\frac{1}{n+1}\; & \{\frac{\frac{\log^{2}\left(z_{1:n}\right)}{\pi \;\zeta^{3}}\frac{e^{-\frac{\log^{2}\left(z_{1:n}\right)}{2\zeta^{2}}}}{\sqrt{1-e^{-\frac{\log^{2}\left(z_{1:n}\right)}{2\zeta^{2}}}}}}{1-\frac{2}{\pi }\arcsin\;\left(\sqrt{1-e^{-\frac{\log^{2}\left(z_{1:n}\right)}{2\zeta^{2}}}}\right)}-\frac{\frac{\log^{2}\left(z_{n:n}\right)}{2\;\zeta^{3}}\frac{e^{-\frac{\log^{2}\left(z_{n:n}\right)}{2\zeta^{2}}}}{\sqrt{1-e^{-\frac{\log^{2}\left(z_{n:n}\right)}{2\zeta^{2}}}}}}{\arcsin\;\left(\sqrt{1-e^{-\frac{\log^{2}\left(z_{n:n}\right)}{2\zeta^{2}}}}\right)}\\ \; & +\sum_{i=2}^{n}\frac{\frac{\log^{2}\left(z_{i:n}\right)}{2\;\zeta^{3}}\frac{e^{-\frac{\log^{2}\left(z_{i:n}\right)}{2\zeta^{2}}}}{\sqrt{1-e^{-\frac{\log^{2}\left(z_{i:n}\right)}{2\zeta^{2}}}}}-\frac{\log^{2}\left(z_{(i-1):n}\right)}{2\;\zeta^{3}}\frac{e^{-\frac{\log^{2}\left(z_{(i-1):n}\right)}{2\zeta^{2}}}}{\sqrt{1-e^{-\frac{\log^{2}\left(z_{(i-1):n}\right)}{2\zeta^{2}}}}}}{\arcsin\;\left(\sqrt{1-e^{-\frac{\log^{2}\left(z_{(i-1):n}\right)}{2\zeta^{2}}}}\right)-\arcsin\;\left(\sqrt{1-e^{-\frac{\log^{2}\left(z_{i:n}\right)}{2\zeta^{2}}}}\right)}\}=0. \end{array}$$

**Figure 4:**
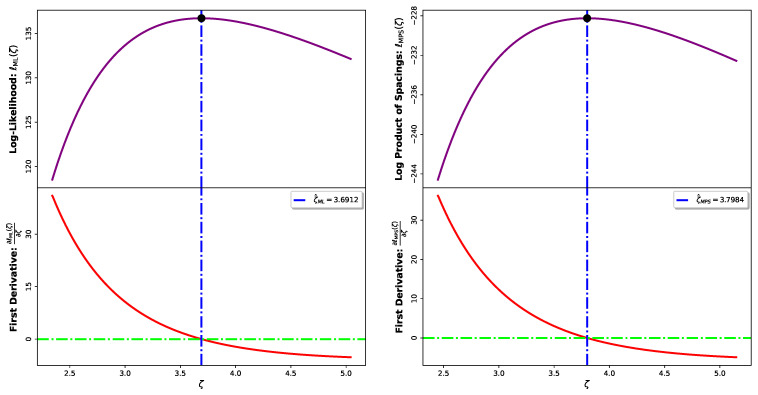
Profile of the log-likelihood and log product of spacings with their corresponding first derivatives. The black points denote the ML and MPS estimators for $\zeta_{\mathrm{true}}=3.50$.

Since Equations (53) and (57) do not admit closed-form solutions, the maximization of $\ell_{\mathrm{ML}}\left(\zeta \right)$ and $\ell_{\mathrm{MPS}}\left(\zeta \right)$ is carried out numerically using the **L-BFGS-B algorithm** in *Python’s SciPy library (Python version 3.10. run on Google Colab)* [24]. Although nonlinear, the objective functions are smooth and locally concave for ζ>0, and the numerical results in Figure 4 suggest unimodality, ensuring convergence to a unique global maximum.

The corresponding estimators of $R_{Z}\left(z;\zeta \right)$ and $h_{Z}\left(z;\zeta \right)$ are then derived by substituting ${\hat{\zeta }}_{\mathrm{ML}}$ or ${\hat{\zeta }}_{\mathrm{MPS}}$ into Equations (5) and (6), respectively, in accordance with the invariance property of ML and MPS estimators [25,26].

### 4.3. Asymptotic Confidence Intervals

Under the standard regularity conditions, the ML estimator ${\hat{\zeta }}_{\mathrm{ML}}$ is asymptotically normally distributed about the true parameter value ζ, i.e., ${\hat{\zeta }}_{\mathrm{ML}}-\zeta \overset{d}{\to }N\;\left(0,I^{-1}\left(\zeta \right)\right)$, where N(·) denotes the normal distribution and $I^{-1}\left(\cdot \right)$ represents the *variance–covariance matrix* of the UASRD parameter obtained from the *Fisher information matrix (FIM)* for ζ, defined as $I\left(\zeta \right)=-\;E\left[\frac{\partial^{2}\ell_{\mathrm{ML}}\left(\zeta \right)}{\partial \zeta^{2}}\right]$. Since the expectation in the *FIM* may not admit a closed-form expression, the observed *FIM* evaluated at the ML estimator is commonly used:

(58) $$\hat{I}\left({\hat{\zeta }}_{\mathrm{ML}}\right)=-\;\frac{\partial^{2}\ell_{\mathrm{ML}}\left(\zeta \right)}{\partial \zeta^{2}}{|}_{\zeta ={\hat{\zeta }}_{\mathrm{ML}}}.$$

Consequently, the asymptotic variance of ${\hat{\zeta }}_{\mathrm{ML}}$ is estimated as $\hat{\mathrm{Var}}\left({\hat{\zeta }}_{\mathrm{ML}}\right)={\hat{I}}^{-1}\left({\hat{\zeta }}_{\mathrm{ML}}\right)$, and a 100(1−γ)% two-sided ACI for ζ is given by

$${\hat{\zeta }}_{\mathrm{ML}}\pm z_{\frac{\gamma }{2}}\sqrt{\hat{\mathrm{Var}}\left({\hat{\zeta }}_{\mathrm{ML}}\right)},$$

where $z_{\frac{\gamma }{2}}$ denotes the upper $\frac{\gamma }{2}$ quantile of the standard normal distribution. For functions of ζ, such as $R_{Z}\left(z;\zeta \right)$ and $h_{Z}\left(z;\zeta \right)$, the *delta method* [27] is employed to approximate the corresponding asymptotic variances:

$$\hat{\mathrm{Var}}\left(R_{Z}\left(z;{\hat{\zeta }}_{\mathrm{ML}}\right)\right)={\left[\frac{\partial R_{Z}\left(z;\zeta \right)}{\partial \zeta }{|}_{\zeta ={\hat{\zeta }}_{\mathrm{ML}}}\right]}^{2}\hat{\mathrm{Var}}\left({\hat{\zeta }}_{\mathrm{ML}}\right)$$

and

$$\hat{\mathrm{Var}}\left(h_{Z}\left(z;{\hat{\zeta }}_{\mathrm{ML}}\right)\right)={\left[\frac{\partial h_{Z}\left(z;\zeta \right)}{\partial \zeta }{|}_{\zeta ={\hat{\zeta }}_{\mathrm{ML}}}\right]}^{2}\hat{\mathrm{Var}}\left({\hat{\zeta }}_{\mathrm{ML}}\right).$$

Accordingly, the 100(1−γ)% ACIs for $R_{Z}\left(z;\zeta \right)$ and $h_{Z}\left(z;\zeta \right)$ are

$$R_{Z}\left(z;{\hat{\zeta }}_{\mathrm{ML}}\right)\pm z_{\frac{\gamma }{2}}\sqrt{\hat{\mathrm{Var}}\left(R_{Z}\left(z;{\hat{\zeta }}_{\mathrm{ML}}\right)\right)}\;\mathrm{and}\;h_{Z}\left(z;{\hat{\zeta }}_{\mathrm{ML}}\right)\pm z_{\frac{\gamma }{2}}\sqrt{\hat{\mathrm{Var}}\left(h_{Z}\left(z;{\hat{\zeta }}_{\mathrm{ML}}\right)\right)}.$$

Analogously, the ACIs for the MPS estimator ${\hat{\zeta }}_{\mathrm{MPS}}$ and its corresponding functions $R_{Z}\left(z;{\hat{\zeta }}_{\mathrm{MPS}}\right)$ and $h_{Z}\left(z;{\hat{\zeta }}_{\mathrm{MPS}}\right)$ are obtained by replacing ${\hat{\zeta }}_{\mathrm{ML}}$ with ${\hat{\zeta }}_{\mathrm{MPS}}$ and using the corresponding observed information from $\ell_{\mathrm{MPS}}\left(\zeta \right)$. These ACIs provide a rigorous measure of uncertainty associated with the parameter estimates and their corresponding functions under both the ML and MPS frameworks [25,26,27].

## 5. Bayesian Inference

In this section, Bayesian methods are employed to estimate the UASRD parameter ζ along with its associated functions $R_{Z}\left(z;\zeta \right)$ and $h_{Z}\left(z;\zeta \right)$. Within the Bayesian paradigm, ζ is regarded as a random variable endowed with a prior distribution. Two prior specifications are considered: an informative Gamma prior and the non-informative Jeffreys’ prior. Specifically, we assume a Gamma prior for ζ with density

(59) $$G\left(\zeta \right)\propto \zeta^{a_{1}-1}e^{-b_{1}\zeta },\;a_{1}>0,\;b_{1}>0,\;\zeta >0,$$

where $a_{1}$ and $b_{1}$ are hyperparameters. For the informative prior, $\left(a_{1},b_{1}\right)$ are chosen by moment matching, equating the prior mean and variance to the empirical moments of ${\hat{\zeta }}_{\mathrm{ML}}$ (or ${\hat{\zeta }}_{\mathrm{MPS}}$) [28]. When prior information is unavailable, we adopt Jeffreys’ prior, $G\left(\zeta \right)\propto \zeta^{-1}$, which corresponds to the limiting case $a_{1}=b_{1}=0$.

### 5.1. Bayesian Estimation Under ML

Let $z_{1},z_{2},\ldots ,z_{n}$ denote a random sample of size *n* from the UASRD with parameter ζ>0, and let $z=(z_{1},\ldots ,z_{n})$. Combining the prior density in Equation (59) with the likelihood function in Equation (51) yields the posterior density of ζ, up to a normalizing constant, as

(60) $$G_{\mathrm{ML}}^{*}\left(\zeta |z\right)=\frac{G(\zeta )\;L(\zeta )}{\int_{0}^{\infty }G\left(\zeta \right)\;L\left(\zeta \right)\;d\zeta }\propto \zeta^{a_{1}}e^{-b_{1}\zeta }\;\prod_{i=1}^{n}\left(-\frac{\log z_{i}\;e^{-\frac{\log^{2}\left(z_{i}\right)}{4\zeta^{2}}}}{\pi \zeta^{2}z_{i}\sqrt{1-e^{-\frac{\log^{2}\left(z_{i}\right)}{2\zeta^{2}}}}}\right).$$

### 5.2. Bayesian Inference Under MPS

An alternative Bayesian estimation procedure is developed under the MPS framework. Let $z_{1:n}<z_{2:n}<\ldots <z_{n:n}$ denote an ordered sample of size *n* from the UASRD with parameter ζ>0, and let $z=(z_{1:n},\ldots ,z_{n:n})$. Combining the prior density in Equation (59) with the product-of-spacings function in Equation (55) yields the posterior density of ζ, up to a normalizing constant, as

(61) $$\begin{array}{cc} G_{\mathrm{MPS}}^{*}\left(\zeta |z\right)\; & =\frac{G(\zeta )\;P(\zeta )}{\int_{0}^{\infty }G\left(\zeta \right)\;P\left(\zeta \right)\;d\zeta }\propto \zeta^{a_{1}-1}e^{-b_{1}\zeta }\{\left[1-\frac{2}{\pi }\arcsin\;\left(\sqrt{1-e^{-\frac{\log^{2}\left(z_{1:n}\right)}{2\zeta^{2}}}}\right)\right]\\ & \;\times \left[\frac{2}{\pi }\arcsin\;\left(\sqrt{1-e^{-\frac{\log^{2}\left(z_{n:n}\right)}{2\zeta^{2}}}}\right)\right]\times \prod_{i=2}^{n}\frac{2}{\pi }[\arcsin\;\left(\sqrt{1-e^{-\frac{\log^{2}\left(z_{(i-1):n}\right)}{2\zeta^{2}}}}\right)\\ & \arcsin\;\left(\sqrt{1-e^{-\frac{\log^{2}\left(z_{i:n}\right)}{2\zeta^{2}}}}\right){\}}^{\frac{1}{n+1}}. \end{array}$$

Bayesian estimation of the UASRD parameter ζ and the associated functions $R_{Z}\left(z;\zeta \right)$ and $h_{Z}\left(z;\zeta \right)$ is conducted under the symmetric squared error loss (SEL) function, $L\left(\zeta ,\hat{\zeta }\right)={\left(\hat{\zeta }-\zeta \right)}^{2}$. Under SEL, the Bayes estimator of any function g(ζ) is its posterior mean,

(62) $${\hat{g}}_{\mathrm{SEL}}\left(\zeta \right)=E\;\left[g(\zeta )|z\right]=\int_{0}^{\infty }g\left(\zeta \right)\;G^{*}\left(\zeta |z\right)\;d\zeta .$$

**Figure 5:**
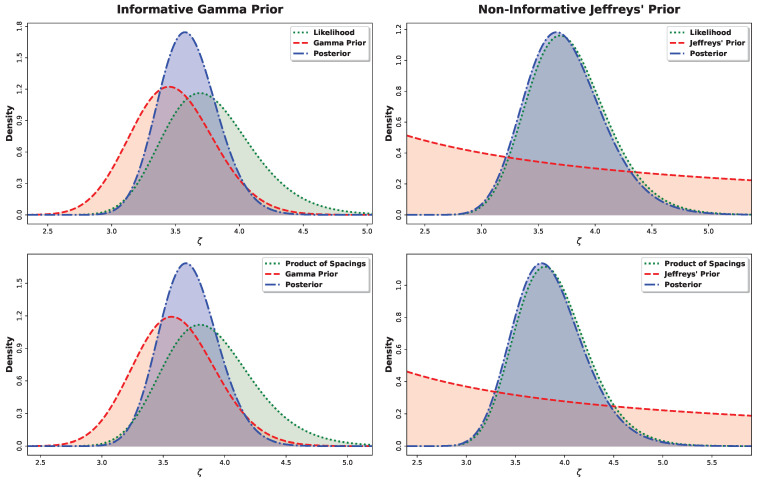
Plots of prior, likelihood (or product of spacings), and posterior distributions at ζ=3.50.

Here, $G^{*}\left(\zeta |z\right)$ denotes the posterior density, which equals $G_{\mathrm{ML}}^{*}\left(\zeta |z\right)$ under the ML-based approach or $G_{\mathrm{MPS}}^{*}\left(\zeta |z\right)$ under the MPS-based approach. Because the posterior distributions in Equations (60) and (61) do not belong to a standard family and are not available in closed form, posterior moments and Bayesian estimates cannot be evaluated analytically. Accordingly, we use Markov chain Monte Carlo (MCMC) methods and employ the Metropolis–Hastings (M–H) algorithm to generate samples from $G_{\mathrm{ML}}^{*}\left(\zeta |z\right)$ or $G_{\mathrm{MPS}}^{*}\left(\zeta |z\right)$. The M–H algorithm is particularly effective here because the posterior distributions are close to normal, as shown in Figure 5, which allows the use of symmetric normal proposal distributions.

Moreover, the acceptance probability ensures convergence to the target posterior [29,30,31]. Based on these samples, we compute Bayesian point estimates and report corresponding highest posterior density (HPD) credible intervals (CRIs). The M–H procedure within Gibbs sampling is implemented as follows:

1. **Input:**
  Starting value $\zeta^{\left(0\right)}=\hat{\zeta }$, proposal variance $\hat{\mathrm{Var}}\left(\hat{\zeta }\right)$, total number of iterations $M^{•}$, burn-in size $M_{0}$, confidence level (1−γ), and posterior density $G^{*}\left(\zeta |z\right)$.
2. Set the iterator to κ←1.
3. **Repeat** for $\kappa =1,\ldots ,M^{•}$:
  (a) Propose a new candidate value $\zeta^{*}∼N\;\left(\zeta^{\left(\kappa -1\right)},\;\hat{\mathrm{Var}}\left(\hat{\zeta }\right)\right),\;\zeta^{*}>0.$
  (b) Evaluate the acceptance probability $\Omega =\min\;\left(1,\;\frac{G^{*}\left(\zeta^{*}|z\right)}{G^{*}\left(\zeta^{\left(\kappa -1\right)}|z\right)}\right).$
  (c) Draw u∼U(0,1).
  (d) **Accept** the proposal if u≤Ω by setting $\zeta^{\left(\kappa \right)}\leftarrow \zeta^{*}$; **otherwise**, keep the previous state, i.e., $\zeta^{\left(\kappa \right)}\leftarrow \zeta^{\left(\kappa -1\right)}$.
  (e) Evaluate $R_{Z}\left(z;\zeta \right)$ and $h_{Z}\left(z;\zeta \right)$ by substituting $\zeta^{\left(\kappa \right)}$ for ζ.
  (f) Increment the iterator: κ←κ+1.
4. Discard the first $M_{0}$ values (burn-in) and keep $\{\zeta^{\left(M_{0}+1\right)},\ldots ,\zeta^{\left(M\right)}\}.$
5. Obtain the Bayes estimator under SEL as ${\tilde{\zeta }}_{\mathrm{SEL}}=\frac{1}{M^{*}}\sum_{\kappa =M_{0}+1}^{M}\zeta^{\left(\kappa \right)},\;\;M^{*}=M^{•}-M_{0}.$
6. Form the (1−γ)100% HPD CRI as follows:
  (a) Arrange the retained values in ascending order as $\zeta_{\left(1\right)}\leq \ldots \leq \zeta_{\left(M^{*}\right)}.$
  (b) For $\kappa =1,\ldots ,\gamma M^{*}$, calculate $\delta \left(\kappa \right)=\zeta_{\left(\kappa +\left\lfloor \left(1-\gamma \right)M^{*}\right\rfloor \right)}-\zeta_{\left(\kappa \right)}.$
  (c) Define $\kappa^{*}=\arg\min_{\kappa }\delta \left(\kappa \right)$.
  (d) The corresponding HPD CRI is $\left(\zeta_{\left(\kappa^{*}\right)},\zeta_{\left(\kappa^{*}+\left(1-\gamma \right)M^{*}\right)}\right).$
7. Repeat Steps (5) and (6) for $R_{Z}\left(z;\zeta \right)$ and $h_{Z}\left(z;\zeta \right)$.

## 6. Monte Carlo Simulation Study

This section presents a Monte Carlo simulation study to evaluate the statistical efficiency of the UASRD model. The analysis focuses on point and interval inference for the parameter ζ and the associated functions $R_{Z}\left(z,\zeta \right)$ and $h_{Z}\left(z,\zeta \right)$, evaluated at the fixed time point Z=0.25.

### 6.1. Simulation Design

To examine estimator performance under varying conditions, several combinations of sample sizes and parameter values are considered. For each configuration, $M_{g}=1000$ independent samples are generated from the UASRD distribution using the inverse transform method in Equation (8). The simulation settings are:

- **Sample Sizes:**
  n=20,40,80,160,320, and 640.
- **True Parameter Values:** ζ=0.25,0.75,2.25,6.75, and 14.

A comparative framework is adopted to study the performance of the ML and MPS estimators, together with their Bayesian counterparts. Classical estimation and the construction of 90% and 95% ACIs are implemented using the **L-BFGS-B algorithm** available in *Python’s SciPy library (Python version 3.10. run on Google Colab)* [24]. Bayesian inference is conducted under informative Gamma priors, with hyperparameters $\left(\mu_{1},\nu_{1}\right)$ specified for each (n,ζ) configuration, and non-informative Jeffreys’ priors to assess prior sensitivity. Posterior summaries under the SEL function, including 90% and 95% HPD CRIs, are obtained via the M–H algorithm described in Section 5. The MCMC procedure is run for $M^{•}=12,000$ iterations, with the first $M_{0}=2000$ iterations discarded as burn-in. The accuracy of the point estimators $\hat{\phi }$, where $\phi \in \{\zeta ,R_{Z}\left(z,\zeta \right),h_{Z}\left(z,\zeta \right)\}$, is evaluated using:

- **Average Estimate (AE):** $\mathrm{AE}\left(\hat{\phi }\right)=\frac{1}{M_{g}}\sum_{i=1}^{M_{g}}{\hat{\phi }}_{\left(i\right)},$ where ${\hat{\phi }}_{\left(i\right)}$ denotes the estimate obtained from the *i*th simulated dataset.
- **Root Mean Squared Error (RMSE):** $\mathrm{RMSE}\left(\hat{\phi }\right)=\sqrt{\frac{1}{M_{g}}\sum_{i=1}^{M_{g}}{\left({\hat{\phi }}_{\left(i\right)}-\phi \right)}^{2}}.$
- **Mean Relative Absolute Bias (MRAB):** $\mathrm{MRAB}\left(\hat{\phi }\right)=\frac{1}{M_{g}}\sum_{i=1}^{M_{g}}\frac{|{\hat{\phi }}_{\left(i\right)}-\phi |}{\phi }.$

Interval estimation is assessed using the 90% and 95% ACIs and HPD CRIs through:

- **Average Length (AL):** ${\mathrm{AL}}_{100(1-\gamma )\%}\left(\hat{\phi }\right)=\frac{1}{M_{g}}\sum_{i=1}^{M_{g}}\left(U_{{\hat{\phi }}_{\left(i\right)}}-L_{{\hat{\phi }}_{\left(i\right)}}\right),$ where γ∈{0.05,0.10} and $L_{{\hat{\phi }}_{\left(i\right)}}$ and $U_{{\hat{\phi }}_{\left(i\right)}}$ denote the lower and upper bounds, respectively.
- **Coverage Probability (CP):** ${\mathrm{CP}}_{100(1-\gamma )\%}\left(\hat{\phi }\right)=\frac{1}{M_{g}}\sum_{i=1}^{M_{g}}I_{\left[L_{{\hat{\phi }}_{\left(i\right)}},\;U_{{\hat{\phi }}_{\left(i\right)}}\right]}\left(\phi \right),$ where I(·) is the indicator function.

### 6.2. Simulation Algorithm

The simulation procedure is implemented in *Python (Python version 3.10. run on Google Colab)* using the autograd and scipy.optimize libraries and proceeds as follows:

**Step 1:**  Specify the true value of ζ and the sample size *n*.
**Step 2:**  Generate *n* observations from U(0,1) and transform them into UASRD samples using $Q_{Y}\left(u;\zeta \right)$.
**Step 3:**  Get the solution of the numerical optimization of ${\hat{\zeta }}_{ML}$ or ${\hat{\zeta }}_{MPS}$.
**Step 4:**  Run the MCMC chains under informative and non-informative priors with a normal proposal distribution.
**Step 5:**  Calculate the AE, RMSE, MRAB, AL, and CP Values of each quantity.
**Step 6:**  Repeat the procedure $M_{g}$ times to arrive at aggregate outcomes.

### 6.3. Simulation Results

**Figure 6:**
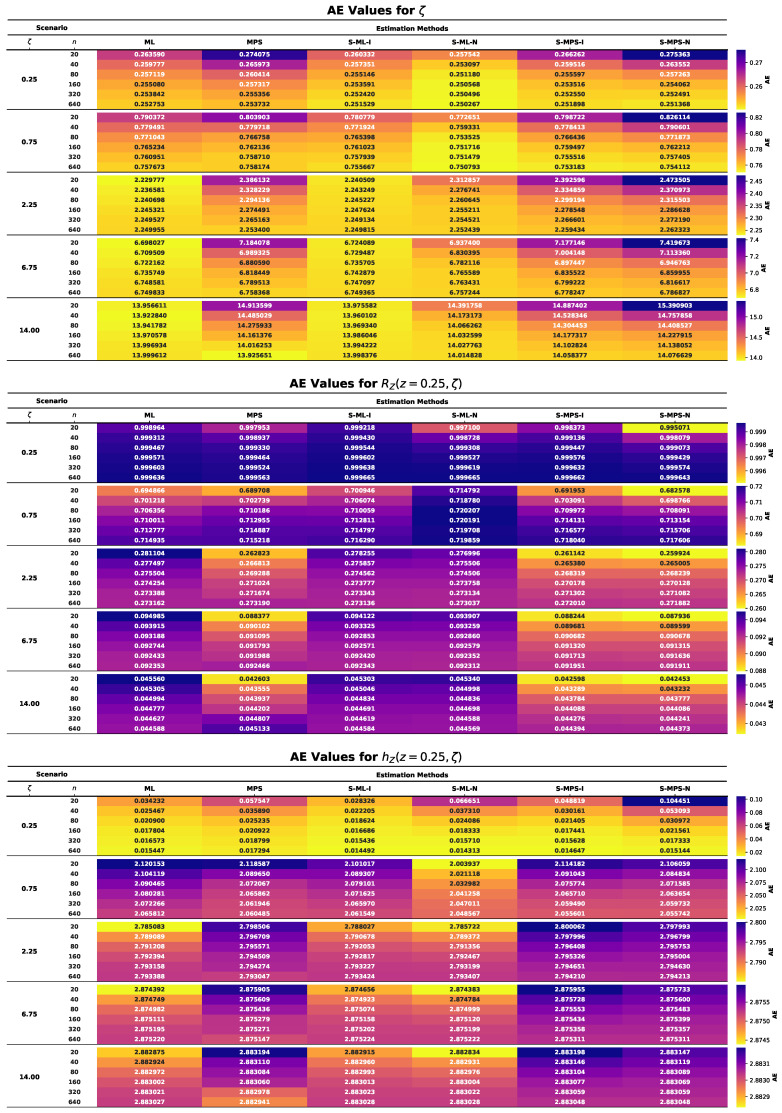
Heatmaps of AE values for ζ (**top**), $R_{Z}\left(z,\zeta \right)$ (**center**), and $h_{Z}\left(z,\zeta \right)$ (**bottom**).

**Figure 7:**
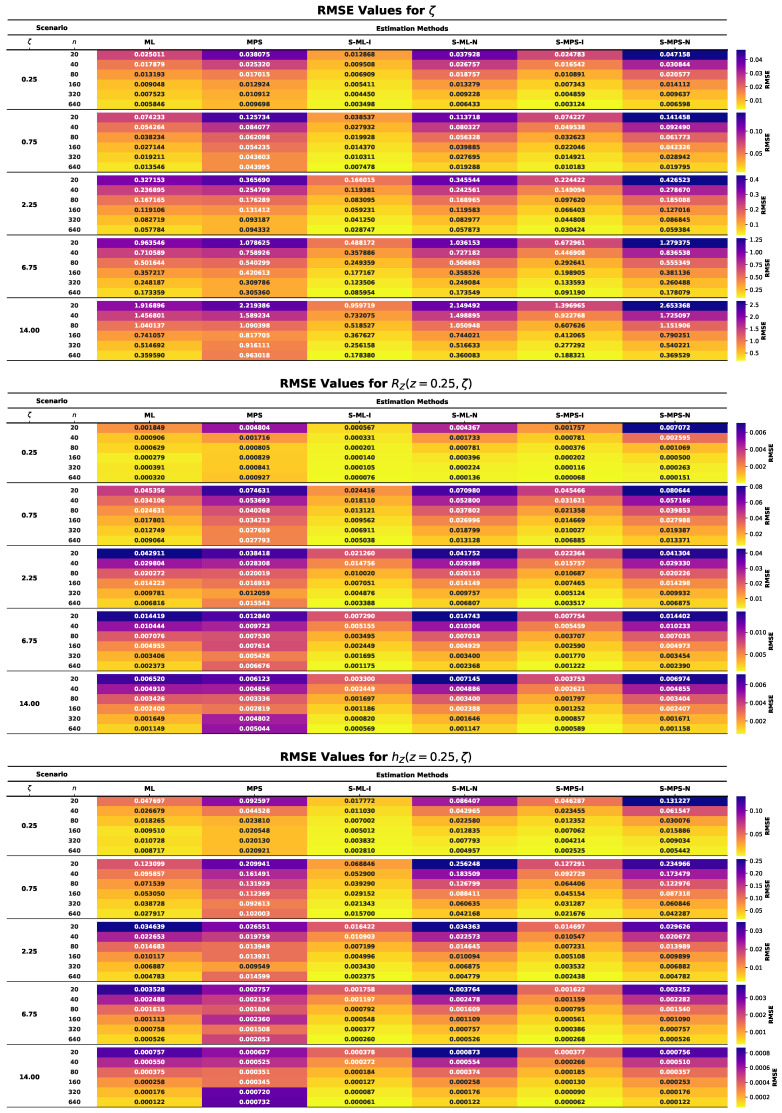
Heatmaps of RMSE values for ζ (**top**), $R_{Z}\left(z,\zeta \right)$ (**center**), and $h_{Z}\left(z,\zeta \right)$ (**bottom**).

**Figure 8:**
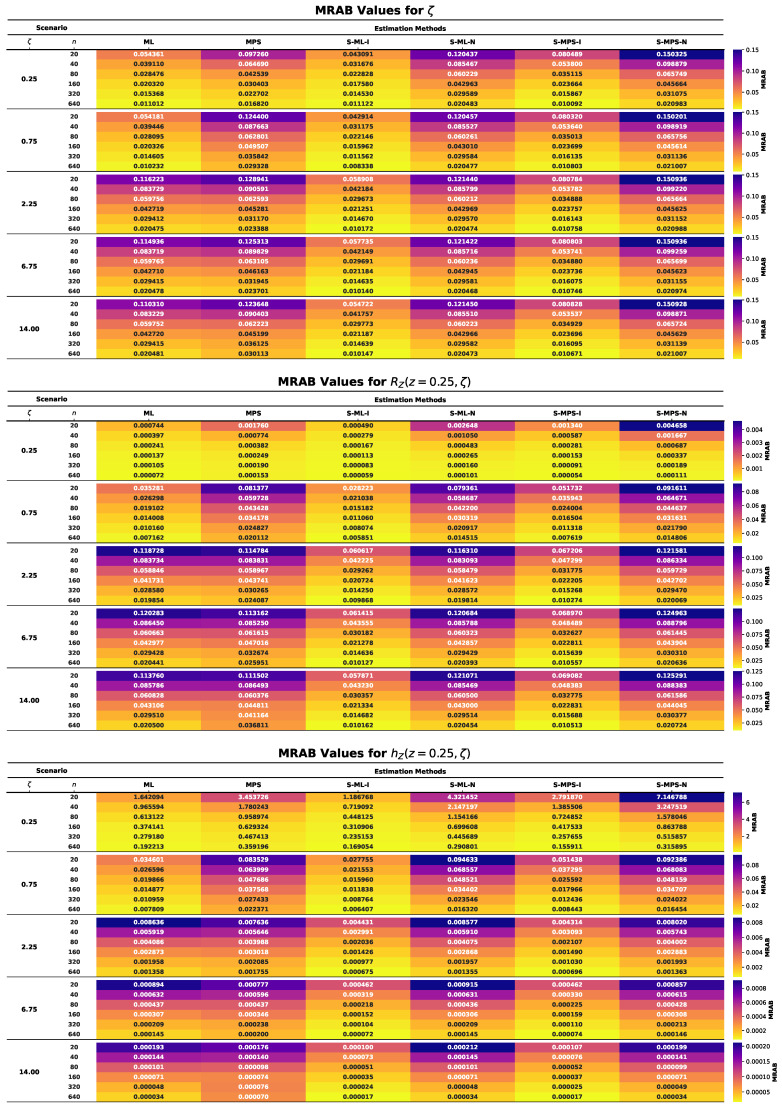
Heatmaps of MRAB values for ζ (**top**), $R_{Z}\left(z,\zeta \right)$ (**center**), and $h_{Z}\left(z,\zeta \right)$ (**bottom**).

**Figure 9:**
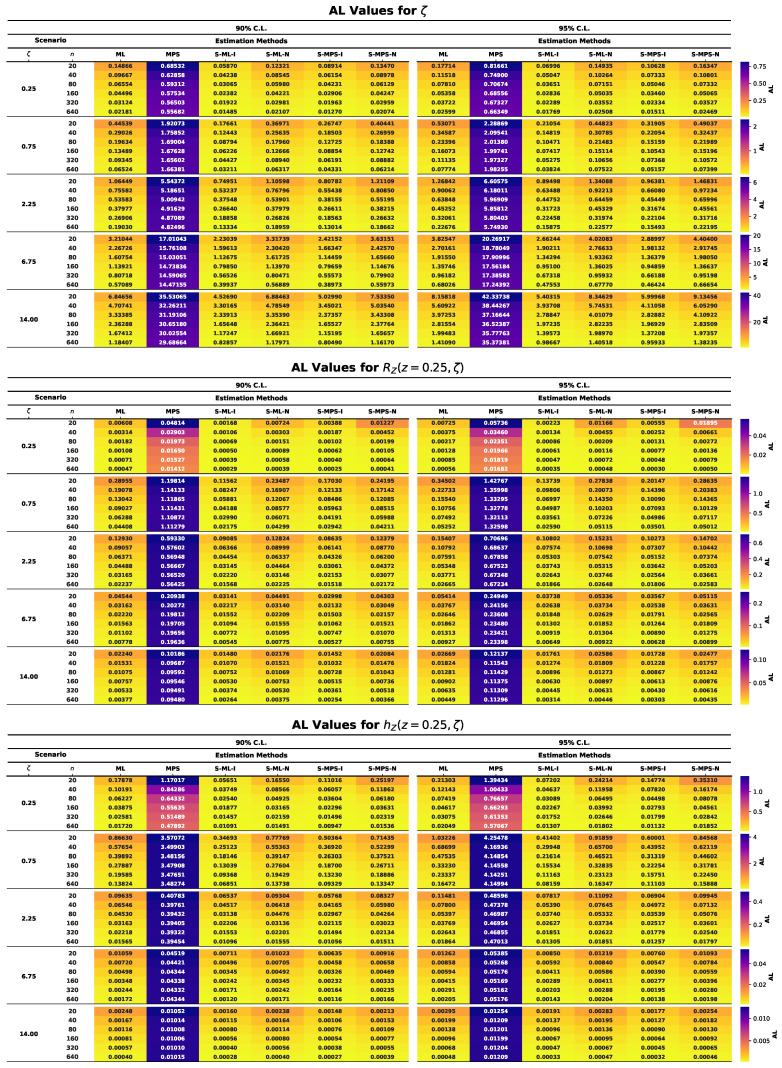
Heatmaps of AL values for ζ (**top**), $R_{Z}\left(z,\zeta \right)$ (**center**), and $h_{Z}\left(z,\zeta \right)$ (**bottom**).

**Figure 10:**
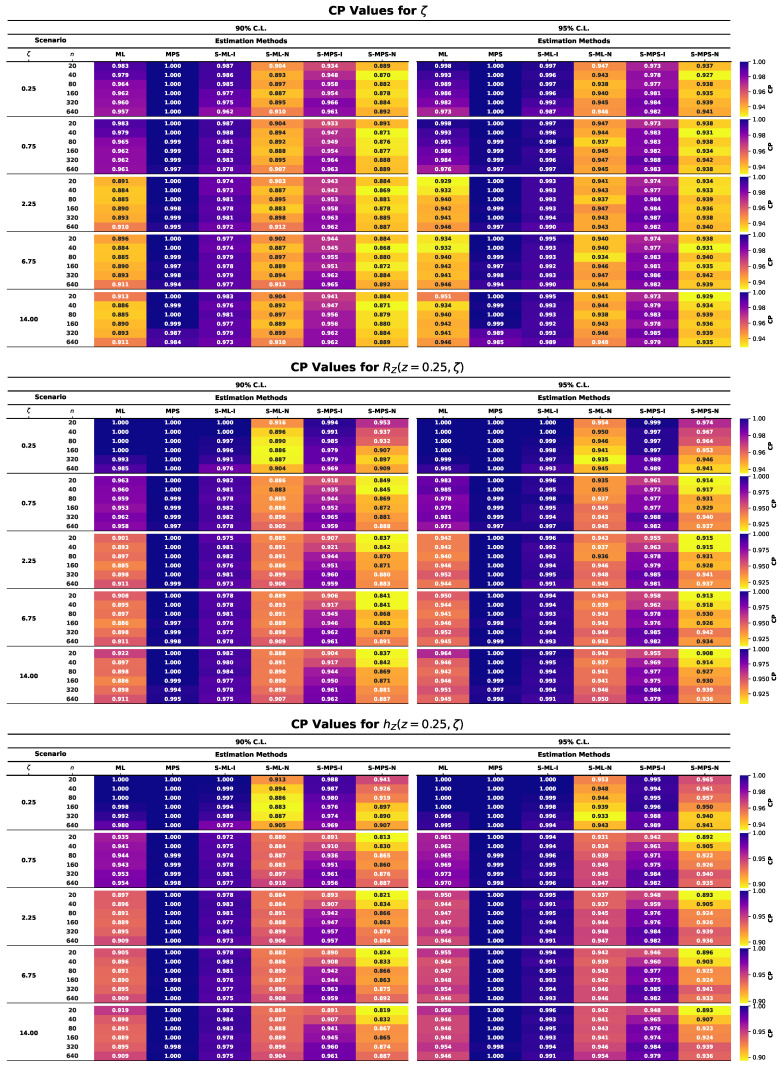
Heatmaps of CP values for ζ (**top**), $R_{Z}\left(z,\zeta \right)$ (**center**), and $h_{Z}\left(z,\zeta \right)$ (**bottom**).

This section presents the results of an extensive MCMC simulation study designed to assess the finite-sample performance of several estimators for the parameter ζ of the UASRD model and the associated reliability measures $R_{Z}\left(z,\zeta \right)$ and $h_{Z}\left(z,\zeta \right)$. The estimators under consideration are classical ML and MPS estimators and their Bayesian counterparts under both an informative and a non-informative prior, i.e., S-ML-I, S-ML-N, S-MPS-I, and S-MPS-N. The findings are summarized using the heatmap visualizations in Figure 6, Figure 7, Figure 8, Figure 9 and Figure 10, allowing comparison of the findings among the sample sizes and different parameter settings. Estimator performance is evaluated using AE, RMSE, MRAB, AL, and CP, which jointly measure point estimation accuracy and interval reliability. The principal findings of the simulation study may be summarized as follows:

1. In all cases, as the sample size *n* increases, the estimators approach the true values, and both RMSE and MRAB decrease, and therefore the estimators are consistent with respect to the estimators of ζ, $R_{Z}\left(z,\zeta \right)$, and $h_{Z}\left(z,\zeta \right)$.
2. The RMSE decreases with increasing sample size. Overall, S-ML-I yields the smallest RMSE and thus the highest precision, followed by S-MPS-I, while MPS and S-MPS-N show comparatively larger variability.
3. The MRAB of each approach reduces as the sample size increases, which is a sign of reduced bias. The fastest decreasing estimates were of informative Bayesian estimators, whereas the bias of MPS-related estimations is relatively higher in small samples.
4. Bayesian estimators using informative priors yield more precise estimations compared to classical methods in small samples based on the values of AE and RMSE, which can be explained by stabilizing the influence of prior information.
5. The AL values decrease with increasing sample size. Informative Bayesian HPD intervals are consistently shorter than classical and non-informative Bayesian intervals, indicating more efficient uncertainty quantification.
6. Increasing the sample size brings CP values closer to the nominal confidence levels. Bayesian informative intervals are mostly the ones nearest to the nominal levels, whereas MPS intervals contain too much, and ML intervals contain too little, when small samples are involved.
7. Informative Gamma priors are better in estimation accuracy and less biased and have a narrower interval width as compared to non-informative ones, which have more variability, especially in small samples.
8. The patterns of performance of the estimators of $R_{Z}\left(z,\zeta \right)$ and $h_{Z}\left(z,\zeta \right)$ are comparable to that of ζ, which indicates stable relative efficiency across the inferential targets.
9. Across most criteria and scenarios, the estimators are ranked in efficiency as
  S-ML-I≳S-MPS-I>ML>S-ML-N>MPS≳S-MPS-N.
10. Bayesian analysis using informative priors, specifically S-ML-I, is the most precise inference on the UASRD, particularly at small and moderate sample sizes, with the difference decreasing with large sample sizes, *n*.

## 7. Data Analysis

This section demonstrates the practical applicability, inferential performance, and modeling flexibility of the proposed UASRD using two real datasets from different applied contexts. The analysis is based on complete observations, which allows a reliable assessment of the model’s ability to represent diverse hazard rate behaviors. The first dataset has 30 observations of the tensile strength of polyester fibers, which were reported in [32]. This amount is the maximum stress that can be applied before failure and is often used in studies of material reliability. The second dataset includes 21 measurements of the computing times required for P3 algorithms to complete specific tasks, as documented in [33], reflecting the stochastic variability inherent in algorithmic execution. The observations are presented as follows:

- **Dataset I: Tensile Strength of Polyester Fibers**
  0.023, 0.032, 0.054, 0.069, 0.081, 0.094, 0.105, 0.127, 0.148, 0.169, 0.188, 0.216, 0.255, 0.277, 0.311, 0.361, 0.376, 0.395, 0.432, 0.463, 0.481, 0.519, 0.529, 0.567, 0.642, 0.674, 0.752, 0.823, 0.887, 0.926.
- **Dataset II: P3 Algorithm Computing Times**
  0.853, 0.759, 0.874, 0.800, 0.716, 0.557, 0.503, 0.399, 0.334, 0.207, 0.118, 0.097, 0.078, 0.067, 0.056, 0.044, 0.036, 0.026, 0.019, 0.014, 0.010.

**Figure 11:**
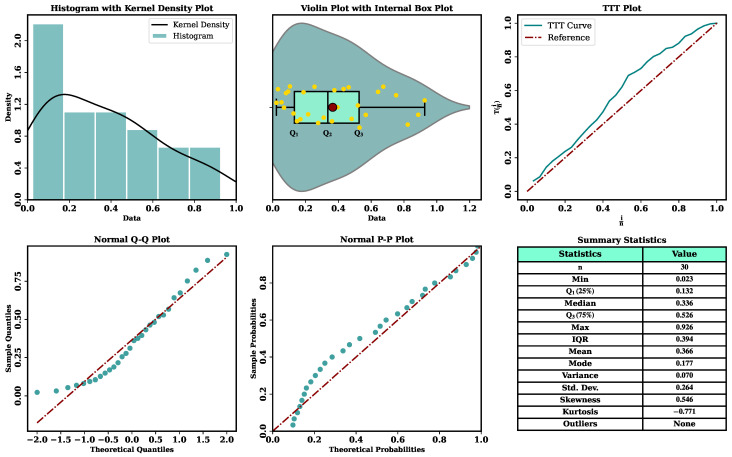
Nonparametric diagnostic plots together with descriptive summary statistics for Data I.

**Figure 12:**
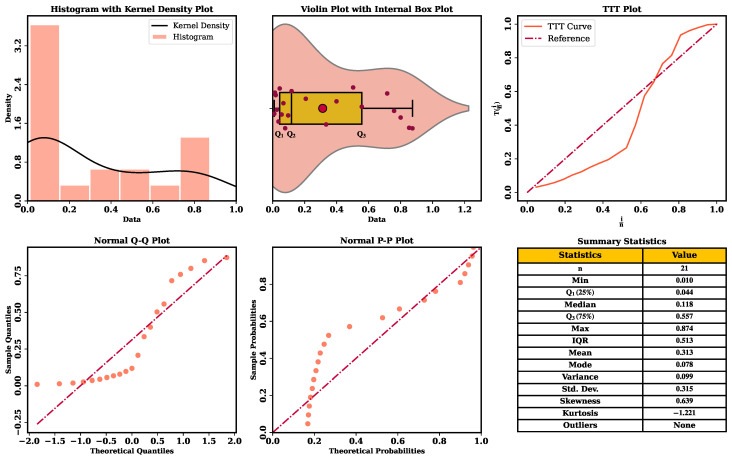
Nonparametric diagnostic plots together with descriptive summary statistics for Data II.

To obtain an initial understanding of the empirical structure of the two datasets, nonparametric graphical tools are employed before conducting formal parametric analysis, as illustrated in Figure 11 and Figure 12.

The summary statistics and graphical analyses of Data I and Data II reveal consistent distributional characteristics. The histogram with kernel density estimation and the violin plot of both datasets show an extreme concentration of values on the lower side of the support, just at the bottom of the range in terms of numbers, especially at the range of values between 0 and 0.2, diminishing in the direction of bigger values. The above trends suggest that there is evidence of skewness in the tails of right behavior since the values of skew and kurtosis are positive and negative, respectively, which denote moderately skewed distributions, which are platykurtic. The TTT plots give inferences about reliability that the hazard behavior is non-linear in accordance with wear-out failure. Additionally, the normal Q-Q and P-P plots show systematic deviations of the reference lines, which proves the fact that the data are not distributed normally. Taken together, the bounded support, evident right skewness, platykurtic shape, and non-constant hazard structure indicate that conventional symmetric models are unlikely to adequately describe the data, thereby motivating the use of the flexible UASRD model for modeling both datasets.

In addition to the proposed UASRD model, several well-known unit distributions are considered for comparison. The unknown parameter of the UASRD is estimated using the ML method, and six alternative models are fitted to both datasets: the Bounded Weighted Exponential Distribution (BWED) [34], the Unit Burr XII Distribution (UBXIID) [35], the Unit Power Distribution (UPD) [36], the Unit Birnbaum–Saunders Distribution (UBSD) [37], the Unit Inverse Gaussian Distribution (UIGD) [5], and the Unit Rayleigh Distribution (URD) [38]. The ML estimates, together with their standard errors (S.E.), the values of −2 times the maximized log-likelihood (−2ℓ), and several penalized likelihood-based model selection criteria, were obtained using the same numerical optimization procedures and convergence criteria to ensure methodological consistency and fairness in the comparison. The Akaike Information Criterion (AIC), the Consistent Akaike Information Criterion (CAIC), the Bayesian Information Criterion (BIC), and the Hannan–Quinn Information Criterion (HQIC) were used to assess model adequacy. In addition, goodness-of-fit was evaluated using empirical distribution function statistics, namely the Anderson–Darling ($A^{*}$), Cramér-von Mises ($W^{*}$), and Kolmogorov–Smirnov (*K*-*S*) statistics, together with the corresponding *K*-*Sp*-values. The results for both datasets are reported in Table 2 and Table 3.

The numerical findings presented in Table 2 and Table 3 are also consistent with the fact that the UASRD provides the lowest values of the information criteria (AIC, CAIC, BIC, and HQIC) when comparing the balance between the model complexity and goodness of fit across the competing unit distributions. Moreover, the UASRD reports the largest *K*-*Sp*-values that exceed 0.05 on both datasets, implying that there is no statistical significance in the empirical and fitted distributions at traditional levels of significance. Overall, these findings support the superior fitting performance of the proposed UASRD model for both datasets.

**Figure 13:**
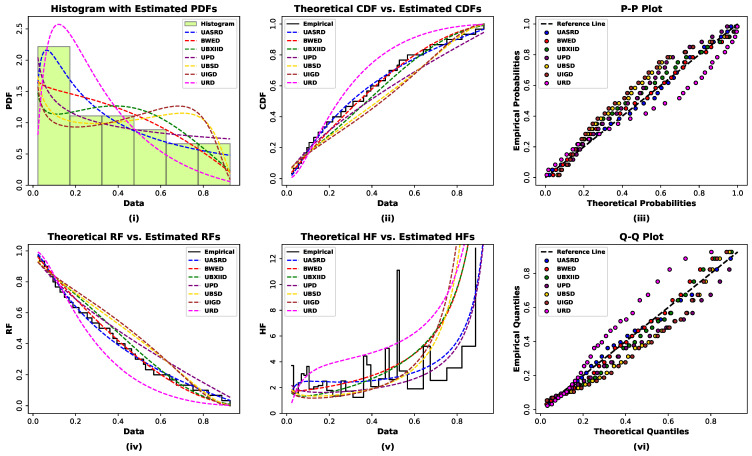
Graphical diagnostic plots for evaluating the goodness-of-fit and comparative adequacy of the fitted distributional models for Data I.

**Figure 14:**
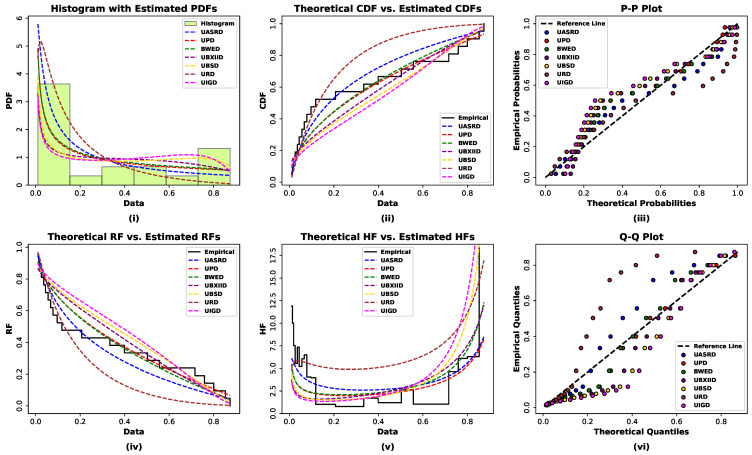
Graphical diagnostic plots for evaluating the goodness-of-fit and comparative adequacy of the fitted distributional models for Data II.

Both sets of data are further supported by the statistical findings in graphical comparisons in Figure 13 and Figure 14. The histograms containing fitted PDFs indicate that the UASRD fits the unit interval of the empirical distribution better than the competing models. The analysis of the empirical and theoretical CDFs shows that the UASRD gives the nearest agreement throughout the support. Likewise, the P-P and Q-Q plots indicate that the empirical distribution is more correlated with the proposed model, especially in the tail areas. The reliability and hazard functions are also estimated to show that UASRD effectively reproduces the observed pattern of survival as well as depicts the non-monotonic, bathtub-like form of hazard structure seen in the data. In general, the graphical diagnostics support the likelihood-based criteria and goodness-of-fit tests and indicate that the UASRD is the most adequate and parsimonious model to represent the two sets of failure-time data.

**Figure 15:**
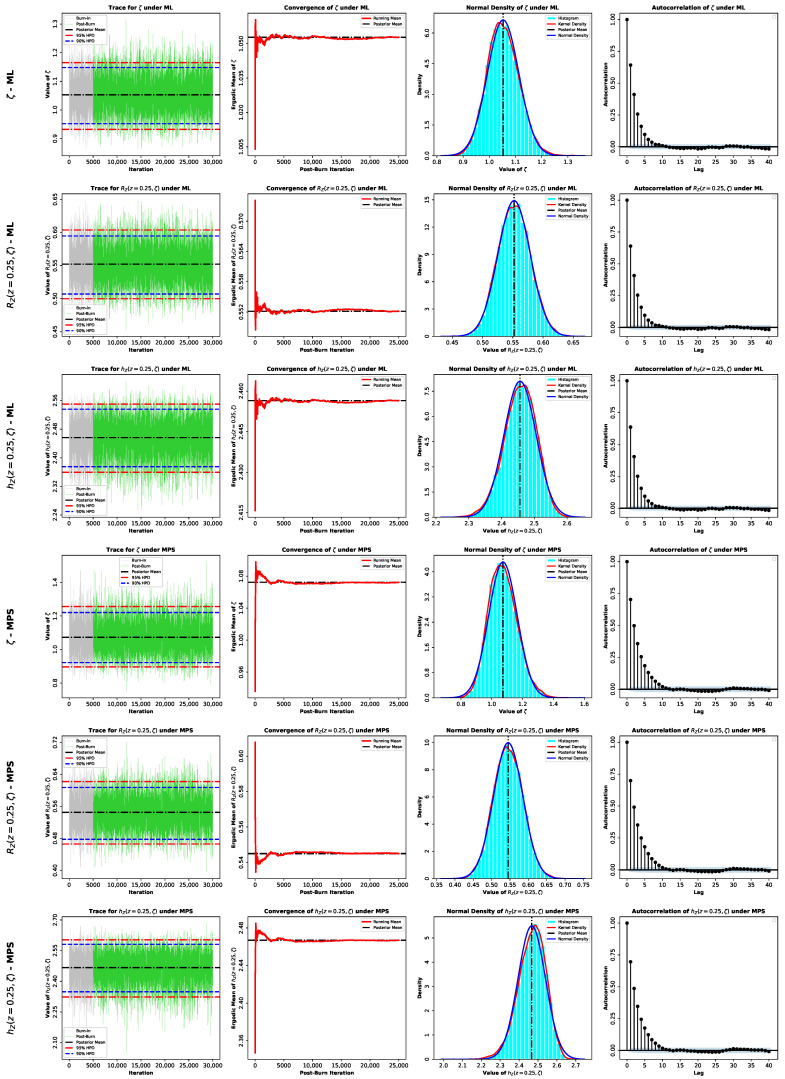
MCMC diagnostic plots for the posterior inference of the model parameter ζ and the reliability measures $R_{Z}\left(z,\zeta \right)$ and $h_{Z}\left(z,\zeta \right)$ evaluated at z=0.25 using an informative Gamma prior for Data I.

**Figure 16:**
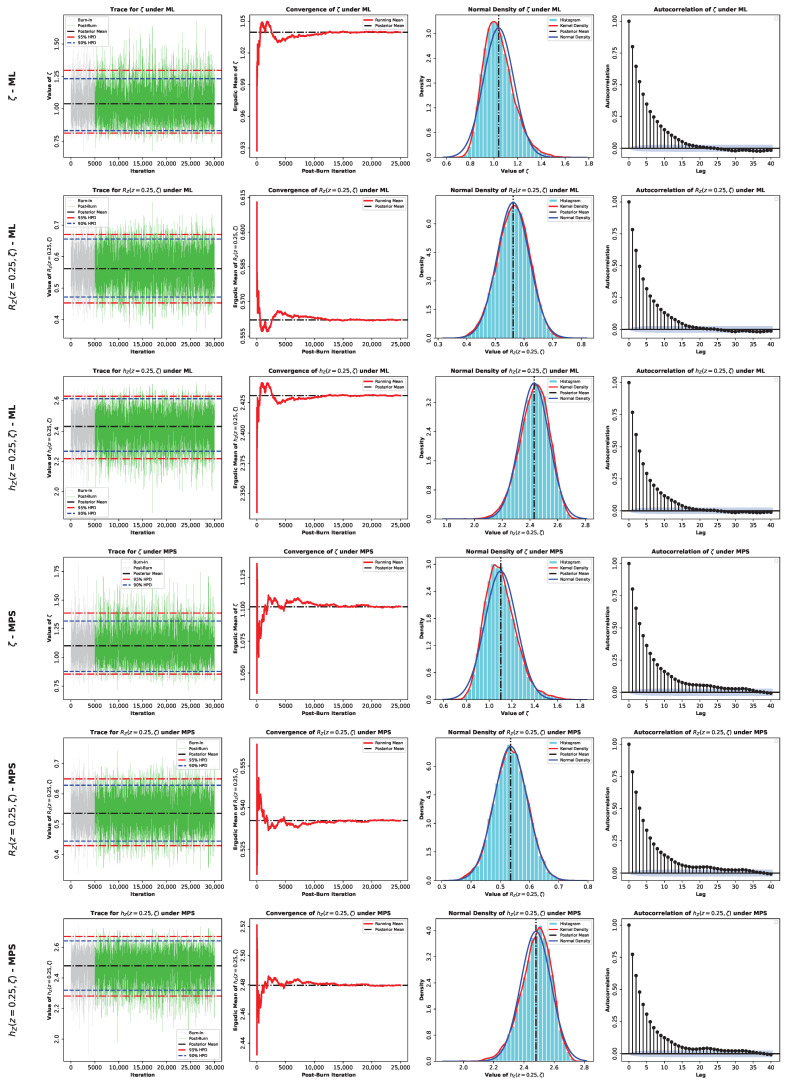
MCMC diagnostic plots for the posterior inference of the model parameter ζ and the reliability measures $R_{Z}\left(z,\zeta \right)$ and $h_{Z}\left(z,\zeta \right)$ evaluated at z=0.25 using a noninformative Jeffery’s prior for Data I.

**Figure 17:**
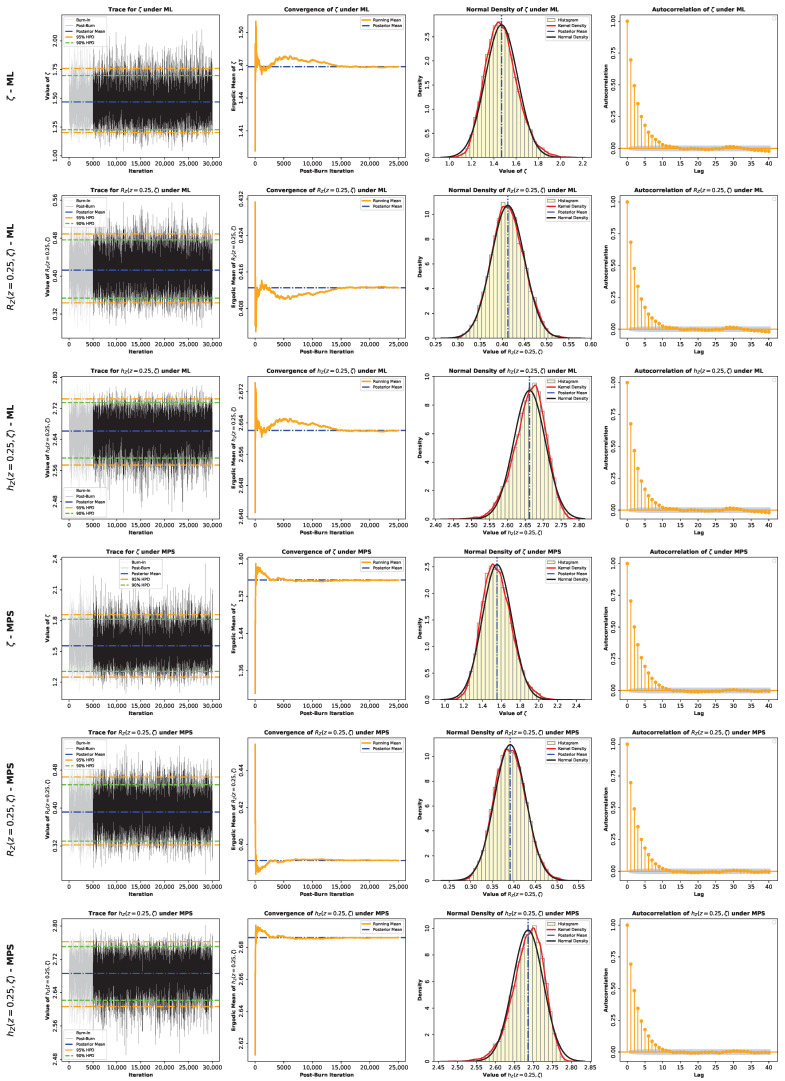
MCMC diagnostic plots for the posterior inference of the model parameter ζ and the reliability measures $R_{Z}\left(z,\zeta \right)$ and $h_{Z}\left(z,\zeta \right)$ evaluated at z=0.25 using an informative Gamma prior for Data II.

**Figure 18:**
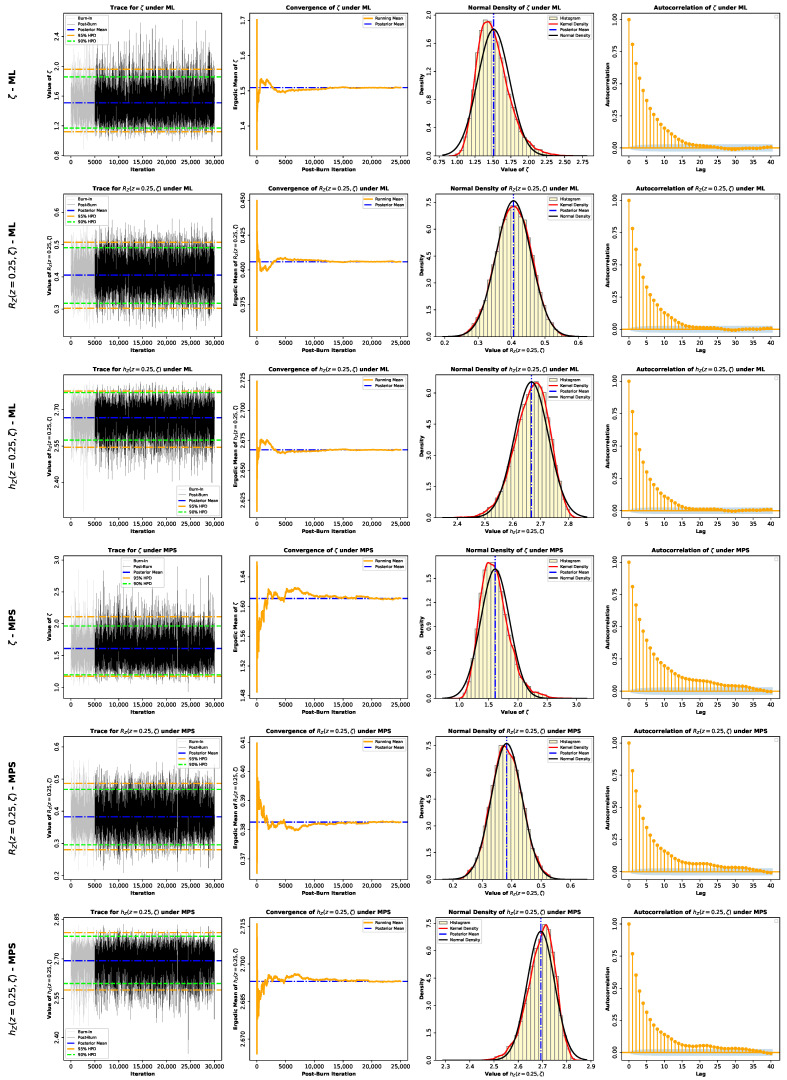
MCMC diagnostic plots for the posterior inference of the model parameter ζ and the reliability measures $R_{Z}\left(z,\zeta \right)$ and $h_{Z}\left(z,\zeta \right)$ evaluated at z=0.25 using a noninformative Jeffery’s prior for Data II.

To further assess the inferential performance of the proposed model, Bayesian estimation of the parameter ζ, as well as the reliability and hazard functions $R_{Z}\left(z,\zeta \right)$ and $h_{Z}\left(z,\zeta \right)$ at z=0.25, was conducted for both datasets. Two prior specifications were considered, namely an informative Gamma prior and the non-informative Jeffreys’ prior, in order to examine the sensitivity of the inference to prior assumptions. Posterior estimation was carried out via MCMC under both the ML and MPS frameworks. The summaries provided in Table 4 and Table 5 show that all the estimation methods are strongly consistent, so the model proposed can be considered stable across both datasets. Bayesian estimators derived under the SEL model have standard errors and credible intervals that are relatively smaller, and this indicates that there is an increased precision of the estimation. The informative prior creates a more concentrated posterior distribution, but the Jeffreys prior creates slightly more widespread ones, though the overall inferences are alike. Furthermore, the MCMC diagnostics in Figure 15, Figure 16, Figure 17 and Figure 18 show a good convergence and sampling efficiency and can be used to support the strength of the Bayesian analysis.

## 8. Conclusions

In this paper, a new unit distribution, called the UASRD, was introduced by applying an exponential transformation to the ASRD. The proposed model possesses closed-form expressions for its principal functions and exhibits flexible shapes for the density, reliability, and hazard rate functions, making it suitable for modeling bounded reliability and proportion data. Several distributional properties and entropy measures were derived to further understand the uncertainty structure and probabilistic behavior of the model. Parameter estimation was developed using the maximum likelihood, maximum product spacing, and Bayesian approaches, and the finite-sample performance of these estimators was examined through Monte Carlo simulation. The practical usefulness of the proposed distribution was illustrated through applications to real datasets. Overall, the UASRD provides a tractable alternative for modeling data supported on the unit interval.

Despite these attractive features, the proposed model has certain limitations. In particular, the presence of only one shape parameter may restrict its flexibility compared with more general multi-parameter unit distributions when modeling highly complex bounded data. Furthermore, while the exponential transformation produces a useful unit model, it may not retain all structural attributes of the original distribution.

Future research may consider extending the UASRD to regression settings, multivariate frameworks, and broader reliability and survival models. It would also be of interest to investigate multi-parameter generalizations and alternative transformation methods to further broaden the applicability of the model.

## Figures and Tables

**Table 1:** Some numerical values for selected descriptive statistics for the UASRD.

ζ	$Q_{1}$	$Q_{2}$	$Q_{3}$	μ	$\sigma^{2}$	$\gamma_{1}$	$\gamma_{2}$	CV	CD
0.03	0.9429	0.9653	0.9833	0.9527	0.0081	−9.5139	97.7301	0.0945	0.0085
0.18	0.7027	0.8090	0.9037	0.7877	0.0217	−1.4319	4.9927	0.1870	0.0275
0.42	0.4390	0.6099	0.7895	0.6030	0.0485	−0.1535	−0.7534	0.3653	0.0805
0.69	0.2586	0.4438	0.6782	0.4671	0.0654	0.2728	−0.9895	0.5474	0.1399
0.95	0.1553	0.3268	0.5859	0.3785	0.0711	0.5615	−0.7997	0.7042	0.1877
1.12	0.1113	0.2675	0.5324	0.3351	0.0716	0.7222	−0.5969	0.7985	0.2137
1.37	0.0682	0.1993	0.4626	0.2852	0.0700	0.9325	−0.2338	0.9274	0.2453
1.63	0.0410	0.1467	0.3996	0.2459	0.0668	1.1271	0.1978	1.0510	0.2716
1.92	0.0232	0.1043	0.3394	0.2123	0.0626	1.3225	0.7220	1.1784	0.2948
2.28	0.0115	0.0683	0.2772	0.1808	0.0573	1.5411	1.4150	1.3241	0.3170
3.15	0.0021	0.0245	0.1699	0.1317	0.0463	1.9937	3.2049	1.6331	0.3513
4.05	3.6 $\times \;10^{-4}$	0.0085	0.1024	0.1019	0.0378	2.3886	5.1562	1.9077	0.3708
5.25	3.4 $\times \;10^{-5}$	0.0021	0.0521	0.0775	0.0298	2.8437	7.8569	2.2270	0.3843
6.10	6.4 $\times \;10^{-6}$	7.6 $\times \;10^{-4}$	0.0323	0.0659	0.0257	3.1325	9.8250	2.4302	0.3893
7.45	4.5 $\times \;10^{-7}$	1.6 $\times \;10^{-4}$	0.0151	0.0529	0.0208	3.5521	13.0380	2.7248	0.3927
8.60	4.8 $\times \;10^{-8}$	4.0 $\times \;10^{-5}$	0.0079	0.0450	0.0177	3.8816	15.8602	2.9550	0.3931
9.85	4.1 $\times \;10^{-9}$	9.2 $\times \;10^{-6}$	0.0039	0.0385	0.0151	4.2185	19.0206	3.1889	0.3919
10.75	7.1 $\times \;10^{-10}$	3.2 $\times \;10^{-6}$	0.0024	0.0348	0.0136	4.4503	21.3587	3.3486	0.3905
11.90	7.4 $\times \;10^{-11}$	8.2 $\times \;10^{-7}$	0.0012	0.0309	0.0120	4.7360	24.4263	3.5441	0.3881
13.50	3.2 $\times \;10^{-12}$	1.2 $\times \;10^{-7}$	5.0 $\times \;10^{-4}$	0.0266	0.0102	5.1181	28.8508	3.8027	0.3843
14.60	3.7 $\times \;10^{-13}$	3.4 $\times \;10^{-8}$	2.7 $\times \;10^{-4}$	0.0242	0.0092	5.3726	32.0036	3.9730	0.3814
15.80	3.5 $\times \;10^{-14}$	8.3 $\times \;10^{-9}$	1.4 $\times \;10^{-4}$	0.0219	0.0083	5.6440	35.5505	4.1529	0.3781
16.90	4.1 $\times \;10^{-15}$	2.3 $\times \;10^{-9}$	7.4 $\times \;10^{-5}$	0.0202	0.0076	5.8881	38.9037	4.3132	0.3751
18.20	3.2 $\times \;10^{-16}$	4.9 $\times \;10^{-10}$	3.6 $\times \;10^{-5}$	0.0184	0.0068	6.1720	42.9964	4.4978	0.3714
19.40	3.1 $\times \;10^{-17}$	1.2 $\times \;10^{-10}$	1.8 $\times \;10^{-5}$	0.0169	0.0062	6.4302	46.9029	4.6640	0.3681
21.10	1.1 $\times \;10^{-18}$	1.6 $\times \;10^{-11}$	7.0 $\times \;10^{-6}$	0.0152	0.0055	6.7912	52.6547	4.8937	0.3635
23.50	9.9 $\times \;10^{-21}$	9.6 $\times \;10^{-13}$	1.8 $\times \;10^{-6}$	0.0132	0.0047	7.2933	61.2244	5.2084	0.3573
26.20	5.0 $\times \;10^{-23}$	4.0 $\times \;10^{-14}$	4.0 $\times \;10^{-7}$	0.0114	0.0040	7.8510	71.5181	5.5517	0.3506
28.40	6.7 $\times \;10^{-25}$	3.0 $\times \;10^{-15}$	1.1 $\times \;10^{-7}$	0.0102	0.0035	8.3017	80.4342	5.8248	0.3455
31.00	4.1 $\times \;10^{-27}$	1.4 $\times \;10^{-16}$	2.7 $\times \;10^{-8}$	0.0090	0.0031	8.8317	91.6016	6.1415	0.3399

**Table 2:** ML estimates with S.E. and goodness-of-fit statistics for the competing unit distributions fitted to Data I.

Model	$\hat{\zeta }\;\left(S.E.\right)$	$\hat{\lambda }\;\left(S.E.\right)$	−2ℓ	AIC	CAIC	BIC	HQIC	$A^{*}$	$W^{*}$	K-S	*p*-Value
**UASRD**	1.0179(0.1206)	−−−	−7.7408	−5.7408	−5.5979	−4.3396	−5.2925	0.1007	0.0141	0.0680	0.9974
BWED	2.2245(6.5893)	0.9504(0.4880)	−5.9148	−1.9148	−1.4703	0.8876	−1.0183	0.2503	0.0274	0.0733	0.9933
UBXIID	1.8465(0.3054)	1.0331(0.2060)	−2.0780	1.9220	2.3665	4.7244	2.8185	0.4999	0.0759	0.0993	0.9008
UPD	0.7254(0.1324)	−−−	−3.4495	−1.4495	−1.3067	−0.0483	−1.0013	0.7238	0.1231	0.1374	0.5755
UBSD	0.8483(0.1444)	1.0772(0.1392)	−1.0101	2.9899	3.4344	5.7923	3.8865	1.1114	0.2120	0.1613	0.3753
UIGD	1.3785(0.3077)	0.9221(0.2381)	2.5566	6.5566	7.0011	9.3590	7.4531	1.4755	0.2769	0.1750	0.2829
URD	0.3482(0.0636)	−−−	0.5438	2.5438	2.6866	3.9450	2.9920	2.3053	0.3054	0.1967	0.1715

**Table 3:** ML estimates with S.E. and goodness-of-fit statistics for the competing unit distributions fitted to Data II.

Model	$\hat{\zeta }\;\left(S.E.\right)$	$\hat{\lambda }\;\left(S.E.\right)$	−2ℓ	AIC	CAIC	BIC	HQIC	$A^{*}$	$W^{*}$	K-S	*p*-Value
**UASRD**	1.4644(0.2056)	−−−	−15.3328	−13.3328	−13.1222	−12.2882	−13.1061	0.6494	0.1000	0.1356	0.7864
UPD	0.5056(0.1103)	−−−	−12.4212	−10.4212	−10.2106	−9.3767	−10.1945	0.6528	0.1125	0.1844	0.4219
BWED	31.618(39.935)	0.5211(0.1152)	−13.2473	−9.2473	−8.5807	−7.1583	−8.7940	0.6993	0.1201	0.1851	0.4174
UBXIID	1.4824(0.2961)	0.8592(0.2144)	−6.5868	−2.5868	−1.9201	−0.4978	−2.1334	1.0572	0.1885	0.2252	0.2038
UBSD	1.0619(0.2396)	1.2620(0.1951)	−11.2616	−7.2616	−6.5950	−5.1726	−6.8083	1.1702	0.2223	0.2380	0.1571
URD	0.1653(0.0361)	−−−	−3.3990	−1.3990	−1.1885	−0.3545	−1.1723	3.8458	0.3225	0.2582	0.1008
UIGD	1.9777(0.6434)	0.8898(0.2746)	−7.7871	−3.7871	−3.1205	−1.6981	−3.3338	1.6139	0.3007	0.2652	0.0858

**Table 4:** Classical and Bayesian estimates of ζ, $S_{Z}\left(z;\zeta \right)$, and $h_{Z}\left(z;\zeta \right)$ evaluated at z=0.25 for Data I.

Par.	Methods	Est.	S.E.	90% C.L.			95% C.L.		
				**Low.**	**Upp.**	**Wid.**	**Low.**	**Upp.**	**Wid.**
ζ	**ML**	1.0179	0.1206	0.8196	1.2162	0.3966	0.7816	1.2542	0.4726
	**MPS**	1.0709	0.1291	0.8586	1.2832	0.4247	0.8179	1.3239	0.5060
	**S-ML-I**	1.0528	0.0601	0.9522	1.1481	0.1960	0.9321	1.1653	0.2332
	**S-ML-N**	1.0396	0.1271	0.8325	1.2334	0.4009	0.8129	1.2978	0.4848
	**S-MPS-I**	1.0724	0.0926	0.9219	1.2201	0.2982	0.8960	1.2559	0.3599
	**S-MPS-N**	1.1020	0.1399	0.8784	1.3174	0.4390	0.8546	1.3876	0.5330
$S_{Z}\left(z,\zeta \right)$	**ML**	0.5670	0.0566	0.4739	0.6600	0.1861	0.4561	0.6778	0.2218
	**MPS**	0.5430	0.0561	0.4507	0.6354	0.1847	0.4330	0.6531	0.2201
	**S-ML-I**	0.5521	0.0267	0.5071	0.5947	0.0876	0.4999	0.6038	0.1038
	**S-ML-N**	0.5620	0.0560	0.4718	0.6558	0.1840	0.4529	0.6705	0.2175
	**S-MPS-I**	0.5450	0.0399	0.4773	0.6072	0.1299	0.4658	0.6219	0.1561
	**S-MPS-N**	0.5353	0.0566	0.4434	0.6282	0.1847	0.4285	0.6495	0.2210
$h_{Z}\left(z,\zeta \right)$	**ML**	2.4308	0.1085	2.2523	2.6092	0.3569	2.2181	2.6434	0.4252
	**MPS**	2.4750	0.0998	2.3108	2.6391	0.3283	2.2794	2.6706	0.3912
	**S-ML-I**	2.4565	0.0492	2.3752	2.5354	0.1601	2.3595	2.5495	0.1901
	**S-ML-N**	2.4308	0.1069	2.2673	2.6137	0.3464	2.2183	2.6296	0.4113
	**S-MPS-I**	2.4669	0.0725	2.3489	2.5809	0.2320	2.3236	2.6027	0.2792
	**S-MPS-N**	2.4796	0.1002	2.3197	2.6422	0.3225	2.2817	2.6708	0.3891

**Table 5:** Classical and Bayesian estimates of ζ, $S_{Z}\left(z;\zeta \right)$, and $h_{Z}\left(z;\zeta \right)$ evaluated at z=0.25 for Data II.

Par.	Methods	Est.	S.E.	90% C.L.			95% C.L.		
				**Low.**	**Upp.**	**Wid.**	**Low.**	**Upp.**	**Wid.**
ζ	**ML**	1.4644	0.2056	1.1262	1.8026	0.6764	1.0614	1.8673	0.8059
	**MPS**	1.5473	0.2220	1.1821	1.9126	0.7304	1.1122	1.9825	0.8704
	**S-ML-I**	1.4687	0.1451	1.2264	1.6980	0.4716	1.2022	1.7598	0.5575
	**S-ML-N**	1.5092	0.2219	1.1695	1.8583	0.6888	1.1201	1.9607	0.8406
	**S-MPS-I**	1.5550	0.1570	1.3051	1.8141	0.5090	1.2493	1.8583	0.6090
	**S-MPS-N**	1.6110	0.2467	1.1987	1.9648	0.7661	1.1754	2.1096	0.9342
$S_{Z}\left(z,\zeta \right)$	**ML**	0.4104	0.0533	0.3228	0.4981	0.1752	0.3060	0.5148	0.2088
	**MPS**	0.3900	0.0522	0.3041	0.4758	0.1716	0.2877	0.4922	0.2045
	**S-ML-I**	0.4125	0.0372	0.3534	0.4764	0.1230	0.3437	0.4888	0.1451
	**S-ML-N**	0.4059	0.0526	0.3182	0.4908	0.1726	0.3029	0.5076	0.2047
	**S-MPS-I**	0.3914	0.0364	0.3302	0.4492	0.1190	0.3224	0.4653	0.1430
	**S-MPS-N**	0.3826	0.0523	0.2956	0.4678	0.1722	0.2804	0.4865	0.2061
$h_{Z}\left(z,\zeta \right)$	**ML**	2.6672	0.0618	2.5655	2.7690	0.2034	2.5461	2.7884	0.2424
	**MPS**	2.6902	0.0565	2.5972	2.7832	0.1859	2.5794	2.8010	0.2216
	**S-ML-I**	2.6621	0.0441	2.5923	2.7353	0.1430	2.5742	2.7450	0.1708
	**S-ML-N**	2.6672	0.0610	2.5749	2.7714	0.1965	2.5451	2.7776	0.2325
	**S-MPS-I**	2.6861	0.0404	2.6215	2.7504	0.1290	2.6066	2.7624	0.1558
	**S-MPS-N**	2.6931	0.0565	2.6060	2.7860	0.1800	2.5815	2.8002	0.2188

## Data Availability

Data are contained within the article.
